# Shrinking sizes of trout and salamanders are unexplained by climate warming alone

**DOI:** 10.1038/s41598-024-64145-x

**Published:** 2024-06-13

**Authors:** Ivan Arismendi, Stanley V. Gregory, Douglas S. Bateman, Brooke E. Penaluna

**Affiliations:** 1 Department of Fisheries, Wildlife, and Conservation Sciences, Oregon State University, Nash Hall 104, Corvallis, OR 97331 USA; 2 Department of Forest Engineering, Resources and Management, College of Forestry, Oregon State University, 210A Snell Hall, Corvallis, OR 97331 USA; 3 USDA Forest Service, Pacific Northwest Research Station, 3200 SW Jefferson Way, Corvallis, OR 97331 USA

**Keywords:** Ecology, Climate-change ecology, Freshwater ecology, Riparian ecology

## Abstract

Decreases in body sizes of animals related to recent climate warming can affect population persistence and stability. However, direct observations of average sizes over time and their interrelationships with underlying density-dependent and density-independent processes remain poorly understood owing to the lack of appropriate long-term datasets. We measured body size of two species common to headwater streams in coastal and Cascades ecoregions of the Pacific Northwest of North America over multiple decades, comparing old-growth and managed forests. We found consistent decreases in median length of Coastal Cutthroat Trout *Oncorhynchus clarkii clarkii,* but a coexisting species, the Coastal Giant Salamander *Dicamptodon tenebrosus*, appears to be more resilient to size changes over time. Based on observed trends, adult trout have decreased in length by 6–13% over the last 30 years. Length decreased more in larger compared to smaller animals, suggesting that these effects reflect changes in growth trajectories. Results from a model-selection approach that included hydroclimatic and biological information as covariates in one of our study ecoregions demonstrated that stream temperature alone did not explain observed length reductions. Rather, a combination of density-dependent (animal abundances) and local density-independent factors (temperature, habitat, and streamflow) explained observed patterns of size. Continued decreases in size could lead to trophic cascades, biodiversity loss, or in extreme cases, species extirpation. However, the intricate links between density-independent and density-dependent factors in controlling population-level processes in streams need further attention.

## Introduction

Body size is one of the most obvious characteristics of organisms and its role in shaping the form and function of animals has attracted the attention of scientists for decades^1–3^. Smaller size at age confers intrinsic disadvantages^3^ including lower fitness and fecundity^4^ as well as shortcomings for competitive interactions with conspecifics^5^ and among species^6^. Shrinking size of organisms has been proposed as the third universal ecological response to climate change^7–9^, in addition to shifts in species distributions^10^ and phenology^10–12^. Conceptual models have also been developed to expand investigations of the effects of shrinking sizes from populations to ecosystems^7–9^, but empirical support to evaluate whether smaller sizes are consistent across taxa and ecoregions remains scarce^8,9,13–15^.

To date, the empirical information used to detect temporal changes in size in response to temperature relies on museum collections^8,14^, short-term laboratory experiments^7,16,17^, or harvested species^7,15,18–20^; but see reef fishes^21^. Size data from museum collections are biased by the lack of representativeness of populations^22^, and experimental studies oversimplify whole ecosystem processes that affect size. Size data from harvested or exploited species have inherent biases^8,14,23^ including the selective pressure of harvest on large individuals^24–27^, changes in capture effort, efficiencies, and shifts in management practices over time^28–31^. These additional factors can differentially affect size-specific life histories, especially for migratory species^20,32,33^. Collectively, these issues make it difficult to identify underlying mechanisms responsible for the observed shifts in size^7,8,34^. Hence, long-term data collected simultaneously from multiple species and their environment using consistent methods are crucial not only to test hypotheses about the coherence of responses across taxa and regions, but also to identify specific factors affecting size.

Body size provides a functional link between individual-level processes of behavior and physiology, including metabolism and growth, with higher-level population processes that can explain larger ecological patterns. For example, changes in temperature can lead to accelerated metabolism and growth rates earlier in life, resulting in smaller adults. Warmer environments can alter the distribution of food resources, which can negatively affect growth rates, or it could also reduce water availability leading to reduced habitat size and decrease the amount of resources per-capita, affecting size. A warming climate has been posited as the single factor explaining patterns of change in size in many cases^7,8,18,35^, but see reef fishes^21^. This proposition has been based, in part, on Bergmann’s ecogeographical rule^36^, which states that larger organisms tend to be found in colder environments whereas the opposite occurs in warmer regions. Bergmann's rule, however, does not seem to apply to ectotherms^37^, including freshwater fishes^38^ and amphibians^39^. Hence, a relationship between temperature and size may not be sufficient to explain the greater reductions in size observed in some freshwater species as compared to terrestrial animals^7,14,17^.

In ectotherms, including freshwater species, patterns of long-term changes in size due to warming are also affected by multiple biotic and abiotic factors that are often ignored or unexplored. Among them are the negative relationships between size and population density^40,41^, intra- and interspecific competition^28,30^, thermal dependence of growth and development modulated by site-specific conditions^42^, and combinations of these factors^9^. These biotic and abiotic factors operate at multiple scales^43^ with effects that can be cumulative. Recent evidence shows that additional factors other than climate can explain trends in size of salmonids in both freshwater (local environmental conditions^15^) and marine systems (competition^44^). Hence, overlooking the environmental complexity can potentially risk ignoring essential information needed to develop management actions for adapting to climate change.

Here, we evaluate whether sizes are shrinking under climate change using a long-term time series dataset of Coastal Cutthroat Trout (*Oncorhynchus clarkii clarkii*) in streams draining either old-growth or managed forests from two different ecoregions including the Oregon Coast (1962-2017) and Oregon Cascades (1987-2022), USA. We also test whether Coastal Giant Salamanders (*Dicamptodon tenebrosus*) display shrinking sizes in streams from one of the ecoregions over time (Oregon Cascades 1993–2022). In our study streams, trout have not been exploited by fishing, and the lifespan of both species is long enough to experience seasonal environmental stochasticity. We have long-term information about population abundances and local hydroclimate in one ecoregion, allowing us to develop and test multiple competing models that illustrate how biotic and abiotic factors can influence patterns of body size considering the local ecological context. These models underlie hypotheses based on the literature about potential influences of density-dependent and density-independent factors on size (Table 1). Our findings demonstrate that multi-decadal population studies can provide foundational information for answering complex questions that emerge from ongoing global environmental change.

**Table 1:** Hypothesized density-dependent (DD) and density-independent (DI) factors influencing body size of freshwater vertebrates based on the literature. DD factors include abundance of freshwater vertebrates. DI factors include habitat size, and annual and seasonal metrics of stream temperature^45^ and discharge^46^ that describe the hydrological regimes influenced by both snow and rain in our study region. JJA = June, July, August; DJF = December, January, February.

Category	Abbrev	Description (units)	Influence on size	Reasoning
Animal abundance (DD)	YOY	Abundance of Coastal Cutthroat Trout YOY (#/50 m)	+	Trout YOY offer a potential food source for larger freshwater vertebrates^47,48^
	Trout_Ab	Abundance of Age 1 + Coastal Cutthroat Trout (#/50 m)	−	Density-dependent effect owing to intraspecific or interspecific competition^49–51^
	Salamander_Ab	Abundance of Coastal Giant Salamander (#/50 m)	−	Density-dependent effect owing to intraspecific or interspecific competition^52,53^
Habitat size (DI)	Hab_size_cascade	Mean of maximum depth of cascade habitats within the stream section (m)	+/−	Deeper cascade habitats represent larger habitats (summer refuges) for trout during the low-flow period^49,54^, but are less preferred by salamanders^55,56^
	Hab_size_pool	Mean of maximum depth of pools within the stream section (m)	+/−	Deeper pools represent larger habitats (summer refuges) for trout during the low-flow period^49,54^, but are less preferred by salamanders^55,56^
	Hab_side_channel	Mean of maximum depth of side channels within the stream section (m)	+	Deeper side channels represent larger habitats (summer refuges) during low-flow periods^49,54,57^
Stream discharge^46^ (DI)	Q_var_low	Coefficient of variation in monthly discharge for JJA— daily min time series (none)	+	Higher variation in summer discharge represents pulses of flow that potentially increase insect drift (i.e., food resources)^58^
	Q_reversal	Proportion of the year with negative changes in flow from one day to the next—daily mean time series (none)	−	Higher negative flow reversal represents flow conditions progressively decreasing that will affect habitat size^49,54,57^
	Q_max	Mean annual 1-day discharge—daily max time series (m^3^ s^-1^)	+	Higher flows will extend the floodplain, providing winter refuges resulting in lower metabolic costs^59,60^
	Q_ratio_min	Mean 1-day summer (JJA) discharge divided by annual median discharge—daily min time series (none)	−/+	Higher flow ratios represent more low-flow days in summer, potentially reducing the availability of trout habitats^49,54,57^, but promoting salamander habitats^55,56^
	Q_ratio_max	Mean 1-day winter (DJF) discharge divided by median discharge—daily max time series (none)	+/−	Higher flow ratios represent more high-flow days in winter, potentially increasing the availability of habitats for trout (floodplain)^59,60^, but not for salamanders^55,56^
Stream temperature^45^ (DI)	T_cold_events*	Proportion of days ≤12 °C—daily mean time series (none)	−	Colder days will decrease metabolism resulting in slower growth rates^61^
	T_optima_events*	Proportion of days between 12 and 15 °C (optimal conditions for trout growth)—daily mean time series (none)	+	More days of optimal thermal conditions would result in faster growth rates if food is available^61,62^
	T_winter	Mean 7-day moving temperature DJF—daily min time series (°C)	+/−	Warmer winters will increase metabolic costs^61,63^, but can promote growth if food and refuges are available^60^
	T_summer	Mean 7-day moving temperature (JJA)—daily max time series (°C)	−	Warmer summers will increase metabolic costs in summer^61,63^

*Removed from the model selection approach due to collinearity (|r| > 0.92) with mean winter and summer temperature.

## Material and methods

### Study sites

**Figure 1:**
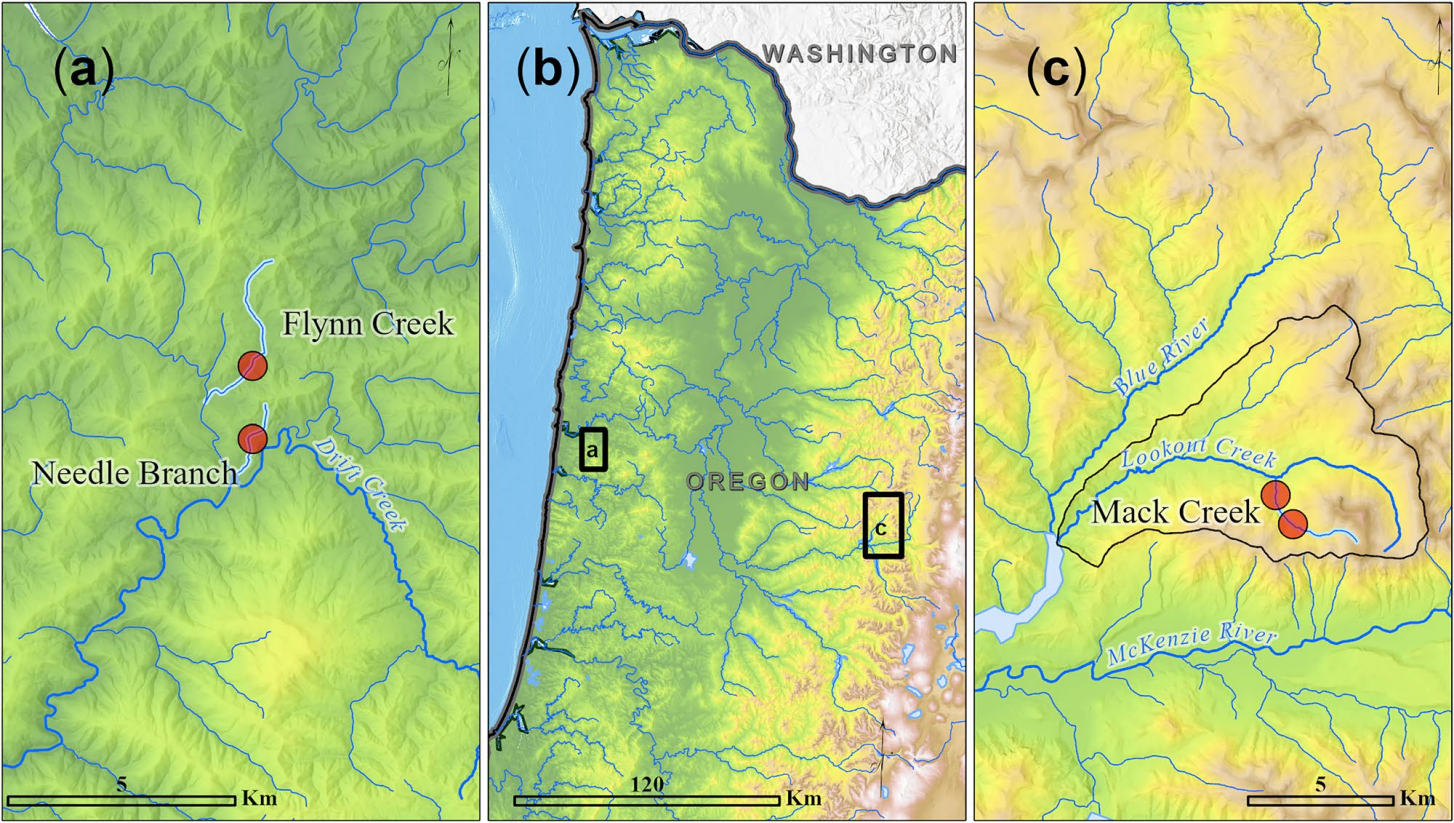
Map of our study sites including (**a**) Flynn Creek and Needle Branch, Coast Range, and (**c**) Mack Creek (old-growth and second-growth sections), Cascade Range. All sites are located in Oregon, USA. Individual body-size measurements of Coastal Cutthroat Trout and Coastal Giant Salamander were collected from our study sites over the last 60 years. [Figure developed Kelly Christiansen (USDA Forest Service, PNW Research Station); Created in ArcGIS PSMFC GIS, Airbus, USGS, NGA, NASA, CGIAR, NCEAS, NLS, OS, NMA, Geodatastyrelsen, GSA, GSI and the GIS User Community].

We used datasets collected from two sections of Mack Creek (old growth and second growth) and two tributaries of Drift Creek (Flynn Creek and Needle Branch) over the last 60 years (Fig. 1). Both Mack Creek and Drift Creek were originally studied as part of large multi-year efforts to assess the effects of forest harvest on freshwater ecosystems and are protected for research purposes^64–67^. Mack Creek is located in the Western Cascade Range of Oregon and Drift Creek lies in the Central Oregon Coast Range, approximately 150 km apart from each other. These drainages are not hydrologically connected. Drift Creek drains directly to the Pacific Ocean approximately 200 km south of the Columbia River estuary, whereas Mack Creek drains via Blue River to the McKenzie River to the Willamette River and ultimately to the Columbia River.

We considered datasets from the old-growth forest section at Mack Creek and Flynn Creek. These sites had no human-related disturbances of forest harvest, land-use changes, commercial, or recreational fishing during the study period and thus, the effects of climate change are isolated (Table 2). Dense old-growth forests including ancient Douglas-fir (*Pseudotsuga menziesii*), Western Redcedar (*Thuja plicata*), and Western Hemlock (*Tsuga heterophylla*) trees of more than 500 years old dominated the old-growth section of Mack Creek. At Flynn Creek, mature forests of approximately 75–155-year-old Douglas-fir and 75–115-year-old trees of Red Alder (*Alnus rubra*) were dominant vegetation in the basin.

**Table 2:** Description of sites (Fig. 1), datasets, and analyses used in this study.

Study site	Flynn Creek, old growth	Needle Branch, second growth	Old growth section of Mack Creek	Second-growth section of Mack Creek
Stream	Flynn Creek	Needle Branch	Mack Creek	Mack Creek
Basin	Alsea River, tributary on Drift Creek	Alsea River, tributary on Drift Creek	McKenzie River, tributary on Lookout Creek	McKenzie River, tributary on Lookout Creek
Basin area (ha)	202	71	580	
Ecoregion	Coast Range	Coast Range	Cascade Range	Cascade Range
Landownership	Siuslaw National Forest, Weyerhaeuser Co	Siuslaw National Forest, Weyerhaeuser Co	H.J. Andrews Experimental Forest, Willamette National Forest	H.J. Andrews Experimental Forest, Willamette National Forest
Land use	Old-growth forest	Second-growth forest (82% of basin clearcut in 1966 with no riparian buffer^66^; 40% of upper portion of watershed clearcut with 15 m riparian buffer in 2009^72^)	Old-growth forest	Second-growth forest (clearcut in 1964)
Dominant trees in forest	75–155-year-old trees of Douglas-fir (*Pseudotsuga menziesii*) and Red Alder (*Alnus rubra*)	Up to 75-year-old trees of Douglas-fir (*Pseudotsuga menziesii*) and Red Alder (*Alnus rubra*)	500-yearold trees of Douglas-fir (*Pseudotsuga menziesii*), Western Redcedar (*Thuja plicata*), Western Hemlock (*Tsuga heterophylla*)	Almost 80-year-old trees of Douglas-fir (*Pseudotsuga menziesii*), Western Redcedar (*Thuja plicata*), Western Hemlock (*Tsuga heterophylla*)
Dominant freshwater vertebrates	Coastal Cutthroat Trout		Coastal Cutthroat Trout and Coastal Giant Salamander	
Animal data*	1962–2017 trout	1962–2017 trout	Annual surveys 1987–2022 trout; 1993–2022 salamanders	
Other freshwater fishes^64,66^	juvenile Coho Salmon (*O. kisutch*), Reticulate Sculpin (*Cottus perplexus*), Pacific Lamprey (*Lampetra tridentata*), and Western Brook Lamprey (*L. richardsoni*)		None	
Hydroclimate**	NA	NA	Mack Creek gage station (daily time series; 1987–2022)	
Habitat data*	NA	NA	Annual surveys (1993–2022)	
Analysis performed in this study	Trends (“Trends in body size of trout and salamander”)	Trends (“Trends in body size of trout and salamander”)	Trends (“Trends in body size of trout and salamander”); Model selection (“Model selection approach to explore factors affecting size at Mack Creek”)	

*In 2020, Mack Creek was not sampled because the H.J. Andrews Experimental Forest was closed owing to a large wildfire. **Daily time series available for the entire study period except for 1989, 1991, 1993, and 2010.

We also considered datasets from streams with an adjacent second-growth forest in Mack and Needle Branch creeks (Table 2). The second-growth forest section of Mack Creek was clearcut in 1964. However, the collection of aquatic vertebrate data started 25 years after timber harvest. Evidence suggests that physical legacy effects of timber harvest should be minimal after 20 years^68–71^. Needle Branch had a clearcut (82% of the basin) in 1966 leaving no riparian buffer^65^ and a second clearcut in 2009 that included approximately 15 m wide riparian buffer on each side of the stream in the upper portion of the study reach (40%; 37 ha) with replanting within 2 years after harvest^72^. These second-growth forest stream sections illustrate a combined effect of climate change and legacies from past forest management.

### Target species

Coastal Cutthroat Trout (*Oncorhynchus clarkii clarkii*) are distributed from Alaska to California^73,74^, whereas Coastal Giant Salamanders (*Dicamptodon tenebrosus*) are present from the coast of southern British Columbia to California^75,76^. These species are tertiary consumers that dominate headwaters in the Pacific Northwest of North America^77^. In its stream-living form, the lifespan of Coastal Cutthroat Trout is 4–5 years (and up to 7–8 years in some cases); individuals are sexually mature around age 1–2, and their home ranges are generally restricted to within 200 m of their birthplace^78^. During seasonal low flow, Coastal Cutthroat Trout prefer deeper pools^79^ with cover availability^54^.

The average Coastal Giant Salamander lifespan is unknown, but animals may live up to 25 years^80^. This species can reproduce as freshwater larvae (neotenes) or as transformed terrestrial adults, with size at maturity between 85 and 115 mm snout-to-vent length^76^. Coastal Giant Salamanders have restricted home ranges (< 30 m) as larvae^81,82^ and adults^83^. Salamander abundances are often negatively associated with wider and deeper streams in western North America^55,84^. Salamanders prefer small pools with slow water velocities^55,56^.

### Animal collection and body size

We evaluated whether there is evidence of shrinking sizes under climate change using individual body length information for Coastal Cutthroat Trout (*Oncorhynchus clarkii clarkii*) from four stream sections in both ecoregions, and Coastal Giant Salamanders (*Dicamptodon tenebrosus*) from two stream sections in one ecoregion (Table 2). All experimental protocols and methods were conducted and approved in accordance with relevant guidelines and regulations from the ethics committee of the Office of Research Integrity at Oregon State University (Institutional Animal Care permits 3720, 4076, 4379, 4796, and 4816).

For Mack Creek, we included trout fork length (FL) and salamander snout-to-vent length (SVL) obtained using standard electrofishing procedures (details in Supporting Information). Trout datasets were available for years 1987–2022, whereas datasets for salamanders were available for years 1993–2022^85^. We distinguished adult trout (Age 1+; FL > 70 mm) from trout young-of-year (YOY; FL ≤ 70 mm) using a visual evaluation of breaks between length classes on length-frequency histograms (Figs. S1, S2). For salamanders, however, we considered all size data owing to the difficulty of determining age based on length (Fig. S2).

Only datasets of trout fork length were available for Flynn Creek and Needle Branch (Fig. 1; Table 2). These datasets were obtained using standard electrofishing procedures during the periods 1962–1974, 1988–1997, and 2006–2017 (details in Supporting Information). Only adult trout (Age 1+) were initially targeted during sampling as questions about climate change and potential shifts in size distributions were not part of the original Alsea Watershed Study. Some inconsistencies in sampling effort resulted in only a few YOY captured for some years. Thus, we aggregated trout YOY data for the entire study period to visually inspect size distributions and separate them from adult trout (FL > 75; Fig. S1).

### Statistical analysis

#### Trends in body size of trout and salamanders

We evaluated temporal trends in length of adult trout and salamanders using the non-parametric Mann–Kendall test for monotonic time series^86^ and the associated Sen’s slope estimator^87^ to estimate the magnitude of trends over time (i.e., mm decade^−1^). This rank-based test is robust for non-normal data, such as time series with outliers and non-linear trends^88^. To perform this analysis, we used the package ‘modifiedmk’^89^ implemented in R ver. 4.2.3. This R package extends the traditional Mann–Kendall test by incorporating modifications to account for serial correlation and offers various functions for trend detection and trend magnitude estimation. We adopted a block bootstrapped^90^ and bias corrected prewhitening^91^ procedure to account for potential serial correlation effects. We evaluated trends using multiple percentiles (i.e., 5th, 10th ⋯ 95th) estimated annually rather than rely on a single central-tendency metric per year (e.g., mean). This approach can correct size-related sampling biases and better describe the typical asymmetry of length distributions (e.g., fewer larger and presumable older animals versus more abundant smaller and presumable younger animals; Figs. S1, S2). The R script used to perform our trend analyses is provided in Supporting Information.

#### Model selection approach to explore factors affecting size at Mack Creek

Due to the absence of additional information at Flynn Creek and Needle Branch Creek, we performed the model section analysis only for Mack Creek where long-term time series of density-dependent (i.e., proportion of YOY trout and abundance of freshwater vertebrates^85^) and density-independent factors (i.e., temperature, discharge, and habitat size^92,93^) were available (Table 2).

We used a multi-model selection procedure as a robust information-theoretic approach for testing multiple hypotheses^94,95^. This analysis allowed us to evaluate the relative roles of density-dependent and density-independent factors affecting size of trout and salamanders (Table 1; Supporting Information). We did not include density-dependent factors related to biomass because time series of body mass were discontinuous. For density-independent factors, we used annual/seasonal metrics of temperature^45^ and discharge^46^ that describe the hydrologic regimes influenced by both snow and rain typical of our study region (Supporting Information). In addition, we added local habitat-size^85^ metrics including the maximum depth of cascades, pools, and side channels within each section of Mack Creek. We performed an additional trend analysis (see “Trends in body size of trout and salamander”) of these covariate factors to explore which of them might change over time. Before performing the model selection, we centered and standardized all density-dependent and density-independent factors (f) to make model coefficients comparable. We tested for potential multicollinearity and removed factors with strong correlation with others within each category (i.e., density-dependent or density-independent) using |r| > 0.7 as recommended threshold^96,97^.

We used generalized linear models (glm) with Gaussian error distributions focusing on main effects and, for simplicity, we did not consider pairwise interactions among factors. We implemented one set of models to predict the annual median length of trout and another set to predict the annual median length of salamanders. In both cases, we considered site (i.e., old-growth and second-growth) as a factor. We fitted all possible models (2f) on each case using the package ‘glmulti’^98^ implemented in R ver. 4.2.3. This R package is a tool for automated model selection and multi-model inference. It employs a genetic algorithm to search through the space of possible models and identifies the most appropriate one based on user-defined criteria. In our case, we separated and ranked all models for trout and salamanders and averaged top-supported models for each case using the Akaike’s Information Criterion corrected for small sample size (AICc). We selected the averaged model (delta AICc < 2) as the best-supported model and used the statistically significant standardized coefficients from conditional models to evaluate size effect among covariates for each case. The full average model assigned zero as the regression coefficient for factors that were not included in the model, whereas conditional averaged model only included the averages of each included factor in the model. The R script used to perform our trend analyses is provided in Supporting Information.

## Results

### Trends in body size

**Figure 2:**
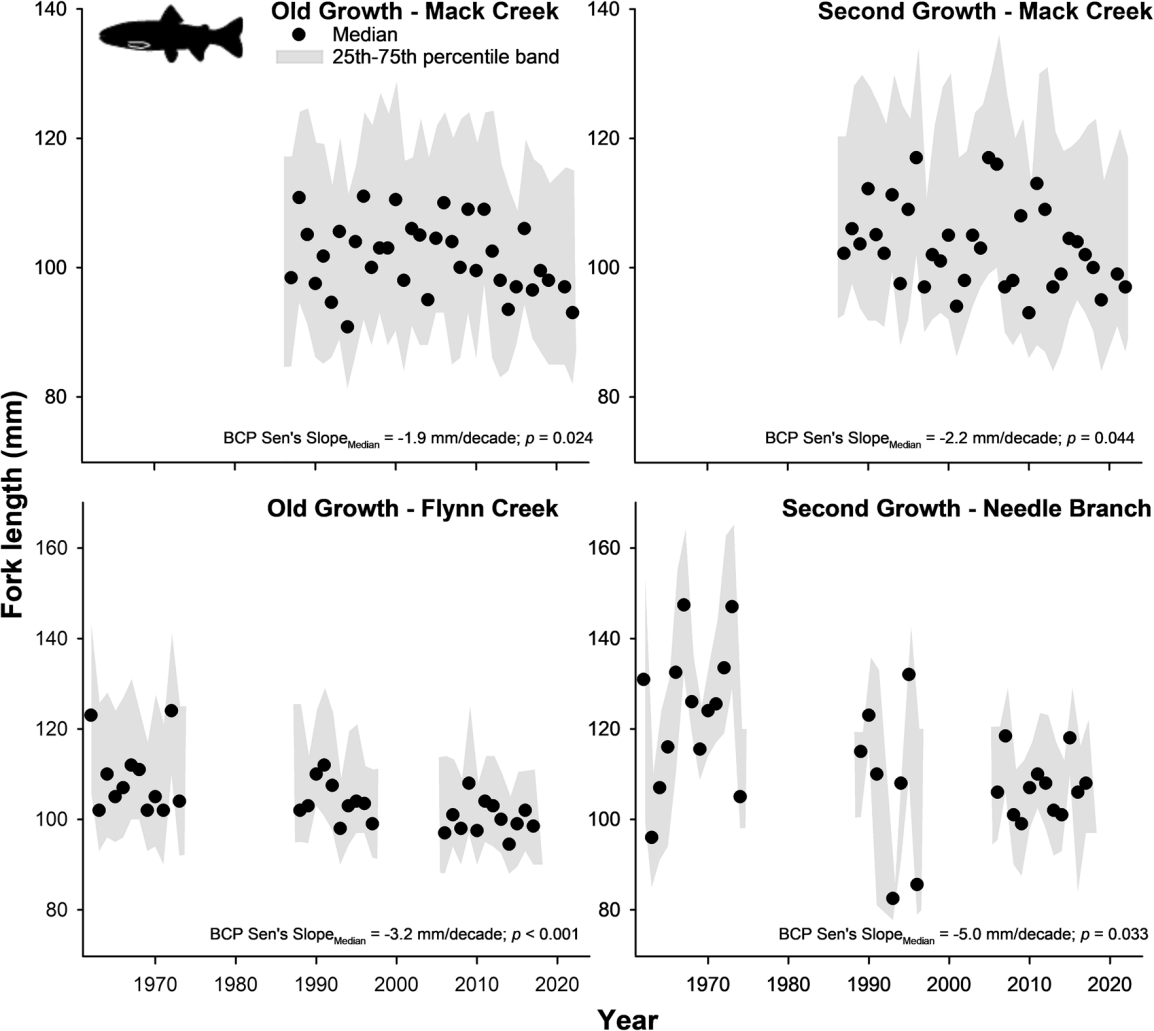
Median fork length (mm) of adult Coastal Cutthroat Trout across sites over time. Bias corrected prewhitened (BCP) Sen’s slope values (mm decade^−1^) represent the rate of change in median size over time (i.e., rate of shrinking size if negative). Shaded area marks 25th–75th percentile band. For detailed statistics, see Tables S1–S4 in Supporting Information.

**Figure 3:**
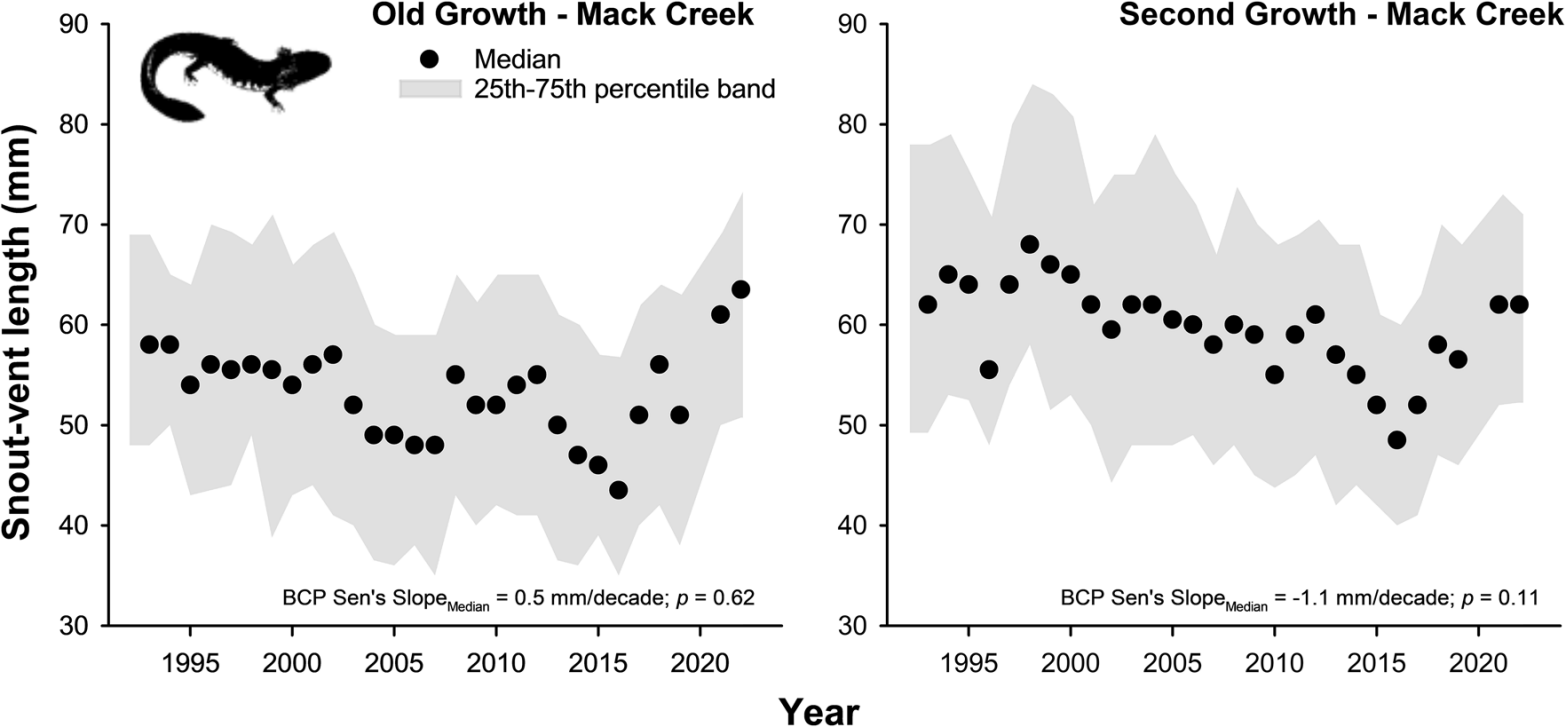
Median snout-vent length (mm) of Coastal Giant Salamander in Mack Creek over time. Bias corrected prewhitened (BCP) Sen’s slope values (mm decade^−1^) represent the rate of change in median size over time (i.e., rate of shrinking size if negative). Shaded area indicates 25th–75th percentile band. For detailed statistics, see Tables S1–S4 in Supporting Information.

**Figure 4:**
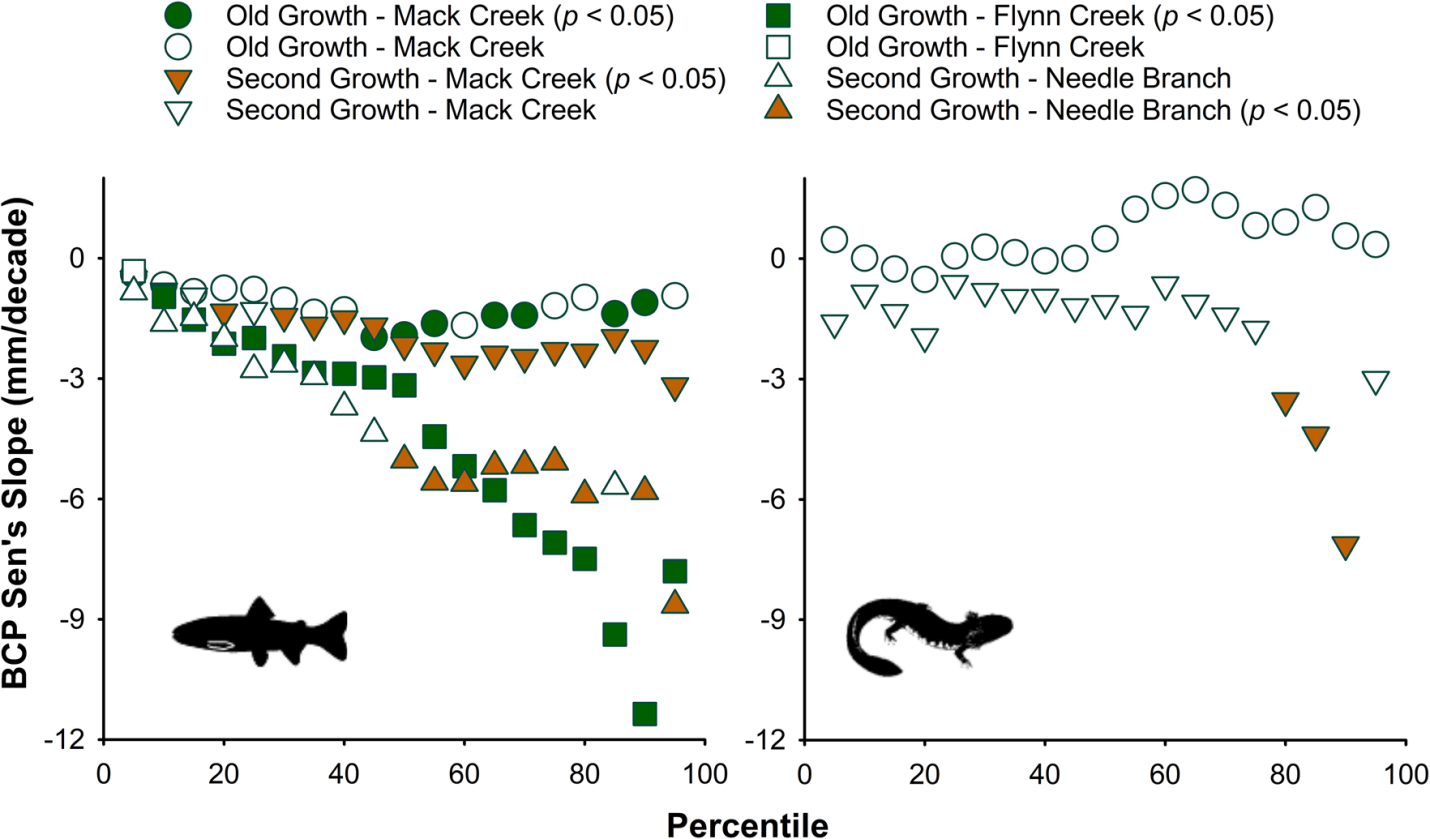
Bias corrected prewhitened (BCP) Sen’s slope values (mm decade^−1^) representing the rate of change in size (i.e., rate of shrinking size if negative) of adult Coastal Cutthroat Trout and Coastal Giant Salamander over time across length percentiles and sites. Filled symbols denote statistically significant trends (Mann–Kendall test *p* < 0.05). For detailed statistics, see Tables S1–S6 in Supporting Information.

We found consistent trends toward decreases in median size over time (between 1.9 and 5 mm per decade) for adult trout at all study sites (Fig. 2; Tables S1–S4), but trends were less consistent for salamanders at Mack Creek (Fig. 3). At the Coast Range sites (Flynn Creek and Needle Branch), Sen’s slope values illustrated that shrinking size rates have occurred almost across the entire length distribution of adult trout (>10th percentile at Flynn Creek; >50th percentile at Needle Branch), yet large and presumably older trout seemed most affected (Fig. 4; Tables S1–S4). Similar patterns of shrinking size rates occurred for adult trout at both sections of Mack Creek (> 45th percentile in the old-growth section; >20th percentile in the second-growth section). In contrast, statistically significant shrinking size rates only occurred for large salamanders (80–90th percentiles) in the second-growth section of Mack Creek (Fig. 4; Tables S5, S6). Our findings were based on annual sampling efforts that resulted on an average of 204 individuals per site and taxon, for a total of 27,244 trout and 12,362 salamander individual observations during the entire study period (Tables S7, S8).

**Figure 5:**
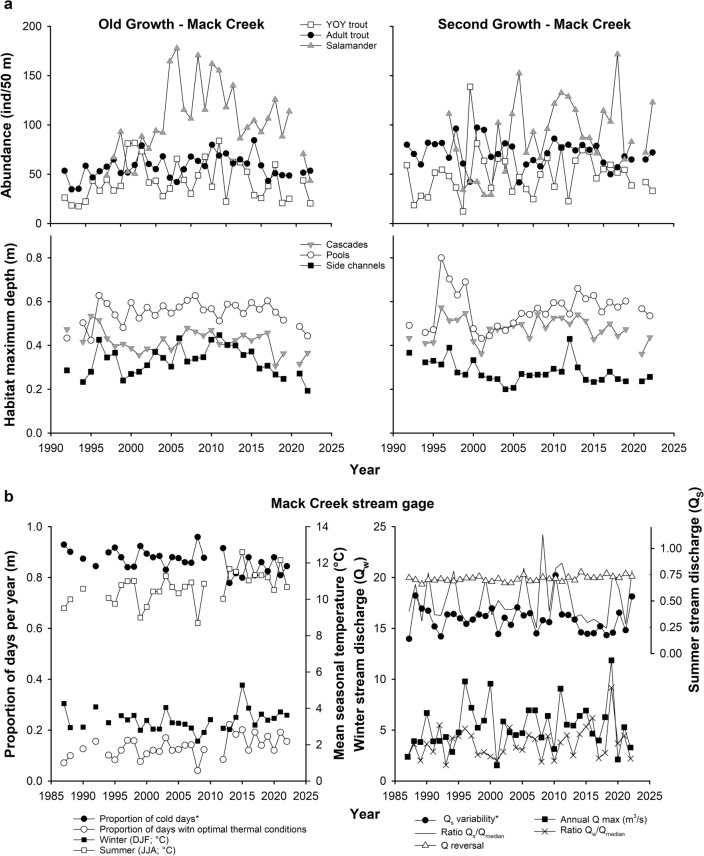
Density-dependent (i.e., abundance of freshwater vertebrates) and density-independent factors (i.e., habitat size, temperature, and discharge) in Mack Creek over time. (**a**) Covariate factors measured during annual sampling events at the old-growth and second-growth sections of Mack Creek. (**b**) Hydroclimatic covariate factors were estimated based on daily time series obtained from a long-term stream gage station located in Mack Creek. See detailed description of these covariate factors in Table 1. *Statistically significant negative trends over time. For detailed trend analyses and statistics, see Table S9 in Supporting Information.

Our temporal examination of covariate factors that potentially affect length of aquatic vertebrates in Mack Creek revealed that only the proportion of cold days (≤12 °C) and the variability of summer discharge have consistently decreased over time (Fig. 5; Table S9). Density fluctuated annually with no apparent long-term trends (Fig. 5a), except for the abundance of salamanders that increased between 1993 and 2008, then gradually decreased between 2008 and 2022 in the old-growth section. Overall, trout (YOY or adults) and salamander abundances were relatively comparable between stream sections, but adult trout seemed slightly more abundant in the second-growth section. Further, density-independent factors related to the size of stream habitats did not change over time, with pools as the deepest habitat units followed by cascades and side channels. Yet, it appeared that cascades were marginally deeper in the second-growth compared to the old-growth stream section. Additional metrics that describe the seasonality of temperature and discharge also fluctuated annually with no apparent long-term trends (Fig. 5b; Table S9). Yet, the warmest winter and summer seasons within our study period coincided with an extreme drought year in 2015.

### Model selection and hypotheses at Mack Creek

**Figure 6:**
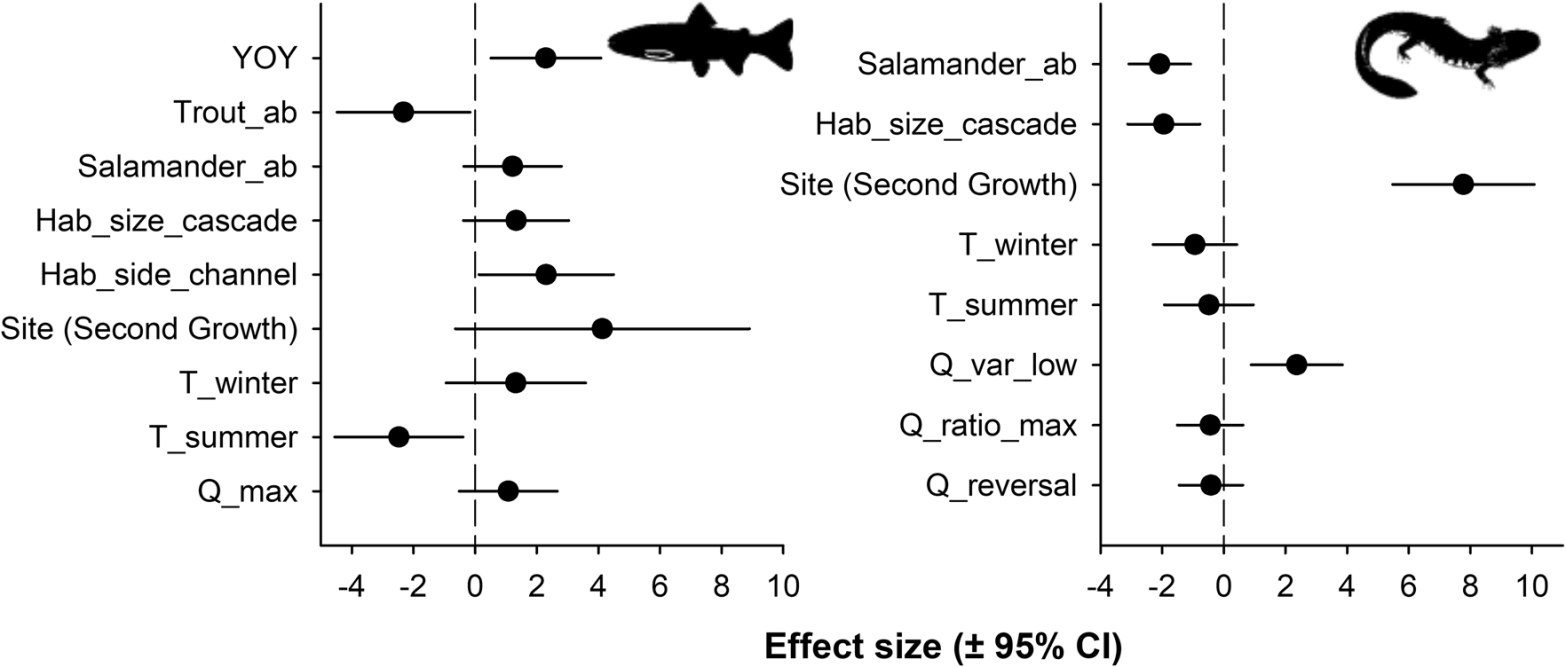
Effect size (standardized values ± 95% CI) of covariate factors from the best-supported model (Tables 3, 4; Tables S10, S11) explaining median size of adult Coastal Cutthroat Trout and Coastal Giant Salamanders in Mack Creek. The best-supported model included both density-dependent (abundance of animals) and density-independent (stream discharge, temperature, habitat size) factors. These biotic and abiotic factors are fully described in Table 1.

Overall, top-supported models (delta AICc < 2) that predicted the median size of adult trout and salamander included a combination of both density-dependent and density-independent factors (Tables 3, 4; Fig. 6). The proportion of cold days (≤12 °C) and proportion of days between 12 and 15 °C (optimal conditions for trout growth) were factors removed from the model selection procedure owing to collinearity (|r| > 0.92) with mean winter and summer temperature. The best-supported model (i.e., conditional average) of adult trout size included the abundance of both trout YOY and adult trout, as well as summer temperature and habitat size (Table 3; Fig. 6; Table S10). As hypothesized (Table 1), warmer summers and more abundant adult trout significantly reduced adult trout size. Also, both deeper side channels and more abundant trout YOY significantly increased the size of adult trout. The predicted effect of temperature was the greatest in the model, followed by adult trout abundance, depth of side-channel habitats, and abundance of trout YOY. Further, the best-supported model of salamander size included, as hypothesized (Table 1), the abundance of conspecifics and depth of cascade habitats, with both negatively affecting salamander size (Table 4; Fig. 6; Table S11). Conversely, the variability of summer stream discharge had a positive effect on salamander size. The median size of salamanders was consistently larger at the second-growth compared to the old-growth stream section of Mack Creek. Lastly, the greatest size effect in this model included site followed by the variability of summer stream discharge and salamander abundance.

**Table 3:** Summary of standardized regression coefficients from the best-supported model of body size (median length) of adult Coastal Cutthroat Trout in Mack Creek, Oregon Cascades.

Model factor	Coefficient estimate	SE	Adjusted SE	Z-value	P-value	Significance level
Full average						
Intercept	101.3	1.29	1.31	77.26	<0.001	***
YOY	2.0	1.13	1.15	1.74	0.081	·
Trout_ab	− 2.2	1.18	1.19	1.84	0.066	·
Hab_size_cascade	0.5	0.84	0.85	0.61	0.540	
T_winter	1.3	1.13	1.16	1.14	0.254	
T_summer	− 2.5	1.03	1.06	2.33	0.020	*
Salamander_ab	0.7	0.84	0.85	0.80	0.426	
Site (second growth)	1.5	2.48	2.49	0.62	0.536	
Hab_side_channel	0.5	1.10	1.11	0.48	0.632	
Q_max	0.4	0.72	0.73	0.57	0.570	
Conditional average						
Intercept	101.3	1.29	1.31	77.26	<0.001	***
YOY	2.3	0.89	0.91	2.52	0.012	*
Trout_ab	− 2.3	1.08	1.10	2.11	0.035	*
Hab_size_cascade	1.3	0.85	0.87	1.52	0.128	
T_winter	1.3	1.13	1.16	1.14	0.254	
T_summer	− 2.5	1.03	1.06	2.33	0.020	*
Salamander_ab	1.2	0.79	0.81	1.50	0.133	
Site (second growth)	4.1	2.39	2.43	1.70	0.090	·
Hab_side_channel	2.3	1.09	1.12	2.07	0.039	*
Q_max	1.1	0.79	0.81	1.32	0.186	

Best-supported model represented the average model among top-supported models (delta AICc < 2; see details in Table S10). The best-supported model included both density-dependent (abundance of animals) and density-independent (stream discharge, temperature, habitat size) factors. These biotic and abiotic factors are fully described in Table 1. Significance levels are represented by ***P < 0.001, **P < 0.01, and *P < 0.05, · = *P* < *0.1* respectively.

**Table 4:** Summary of standardized regression coefficients from the best-supported model of body size (median length) of Coastal Giant Salamander in Mack Creek, Oregon Cascades.

Model factor	Coefficient estimate	SE	Adjusted SE	Z-value	P-value	Significance level
Full average						
Intercept	52.8	0.77	0.79	66.92	< 2e−16	***
Site (second growth)	7.8	1.14	1.17	6.63	< 2e−16	***
Salamander_ab	−2.1	0.50	0.51	4.05	0.000	***
Hab_size_cascade	−1.9	0.58	0.60	3.26	0.001	**
T_winter	−0.9	0.68	0.69	1.35	0.178	
T_summer	−0.5	0.71	0.74	0.66	0.511	
Q_var_low	2.4	0.74	0.76	3.13	0.002	**
Q_ratio_max	−0.1	0.30	0.30	0.30	0.768	
Q_reversal	−0.1	0.28	0.29	0.29	0.776	
Conditional average						
Intercept	52.8	0.77	0.79	66.92	< 2e−16	***
Site (second growth)	7.8	1.14	1.17	6.63	< 2e−16	***
Salamander_ab	−2.1	0.50	0.51	4.05	0.000	***
Hab_size_cascade	−1.9	0.58	0.60	3.26	0.001	**
T_winter	−0.9	0.68	0.69	1.35	0.178	
T_summer	−0.5	0.71	0.74	0.66	0.511	
Q_var_low	2.4	0.74	0.76	3.13	0.002	**
Q_ratio_max	−0.4	0.53	0.55	0.81	0.418	
Q_reversal	−0.4	0.51	0.53	0.78	0.433	

Best-supported model represented the average model among top-supported models (delta AICc < 2). However, models ranked as second and third were also included here and averaged as these models were near the delta AICc threshold (see details in Table S11). The best-supported model included both density-dependent (abundance of animals) and density-independent (stream discharge, temperature, habitat size) factors. These biotic and abiotic factors are fully described in Table 1. Significance levels are represented by *** = *P* < 0.001, ** = *P* < 0.01, and * = *P* < 0.05, · = *P* < *0.1* respectively.

## Discussion

We show empirical support for consistent declines in trout length across ecoregions based on data that span decades, highlighting the importance of long-term ecological research in detecting this gradual shrinking of sizes over time. Based on observed trends, adult trout size reductions over the last 30 years are estimated to be between 6 and 13%. In Mack Creek, trends in length for salamanders are less consistent, suggesting complex responses between coexisting freshwater vertebrates to climate change. Our model-selection approach in Mack Creek offers additional evidence pointing to the possibility that reductions in size might not solely be related to climate warming, but also include a combination of density-dependent and density-independent factors, emphasizing the relevance of local ecological contexts. Our findings provide valuable insights into the responses of freshwater taxa to climate change that would not be possible without the careful maintenance and attention given to the consistent collection of these data as part of long-term research programs.

### Consistent shrinking sizes of trout across sites, but not in stream salamanders

The consistent decline in sizes of trout from relatively pristine systems provides empirical support to the idea of shrinking size as an ecological response to climate change. To date, only a few examples from natural settings for large freshwater predators^7,15,17^ have been examined to test the hypothesis of smaller size of animals related to climate change in freshwaters; our study helps fill this information gap. Yet, there is conflicting evidence about whether a decline in size has occurred in salamanders with decreases^63,99^, increases^99^, and no apparent trends in size over time, except for larger animals (this study). Also, increases and decreases in size of salmonids and other exploited fishes illustrate complex interactions among size-selected harvest, environmental conditions, and other human influences such as hatchery-origin stocks^15,30,44,100^. Human-related influences on sampling and exploited animals can obscure trends in size.

Reductions in size of trout appear to reflect changes in growth trajectories, with greater magnitudes occurring for larger and likely older animals. In our study region, the increase in the frequency and intensity of droughts over time^101^ can differentially affect annual growth rates of larger trout, as shown after the occurrence of such extreme events^50^. Although the magnitude of decreases in size of adult trout is relatively small (mm per decade), trends are remarkably consistent and comparable in magnitude to other freshwater organisms^17^. In addition, the reduction in size we observed for larger salamanders (80th–90th size percentiles) in the second-growth section of Mack Creek is almost twice the magnitude reported for terrestrial salamanders^63^. More research is needed to understand the differential adaptation between coexisting species^21^ across ecoregions and to disentangle the role that local contexts play in modifying rates of shrinking sizes over time.

### Density-dependent and density-independent processes differentially affect size of trout and stream salamanders in Mack Creek

Findings from our model selection for Mack Creek demonstrate that the sizes of trout and stream salamanders are simultaneously influenced by density-dependent and density-independent processes. Therefore, reductions in size cannot be attributed to climate warming alone and are consistent with recent findings for salmonids in freshwater^15,102^ and marine systems^44^. Body size of an amphibian population varies with abundance and not climate^103^, emphasizing the importance of density-dependent factors. Considering how density-dependent and density-independent factors might influence the size of these freshwater vertebrates, our best-supported models highlight the importance of considering local ecological contexts to explain observed declines in size.

Density-dependent factors in the best-supported model predicting size of adult trout include positive associations with the abundance of trout YOY, and negative associations with the abundance of adult trout. For salamander size, the best-supported model includes negative associations with the abundance of salamanders. Both Coastal Cutthroat Trout and Coastal Giant Salamanders are generalist opportunistic feeders^48,78,104^. Headwater streams in the Pacific Northwest have low levels of primary and secondary productivity^105^. Thus, trout YOY represent a potential food source for large trout during summer^47^. Growth in salmonids is predominantly regulated by density-dependent factors, even at low population densities^50,51,106^ owing to competition for food and space^107^. Both trout and salamanders^52^ also exhibit territorial behavior^107^. Hence, density-dependence can decrease food availability per capita resulting in decreases in size of these freshwater vertebrates.

Density-independent factors are also incorporated in the best-supported models that predict size in adult trout and salamanders. Specifically, warmer summers negatively affect trout size. In Mack Creek, maximum stream temperatures are far below the critical thermal tolerances reported for Coastal Cutthroat Trout^62^, but warmer summers can increase metabolic costs^61^ with negative effects on growth^50^. In addition, our model shows that deeper side channels positively affect trout size. This is consistent with trout preferring relatively lentic and deeper habitats^57^ where there is a greater provision of in-stream cover^54^. Further, density-independent factors that describe seasonal stream discharge and local habitat size are also included in the best-supported model that predicts size of salamanders. Specifically, the higher variability of summer stream discharge exerts a positive influence, whereas deeper cascade habitats negatively influence salamander size. Higher variability of summer stream discharge would offer greater food opportunities owing to increases in total invertebrate-drift load during higher-flow events^58^. The negative association between size and depth of cascades aligns with stream salamander abundances as being inversely related to wider stream channels in western North America^55,84^ and their preference for smaller pool habitats with slower water velocities^55,56^.

### Trends in size in the context of local forest management and climate change

Size reductions in adult trout differ in magnitude across our study sites, suggesting that the combination of local factors and climate change can modulate rates of size decrease over time. For example, median trout size decreases slightly more in sites with a legacy of forest harvest than in sites dominated by old-growth forests, evidence that relatively pristine ecosystems could buffer against the effects of climate change. The buffering capacity against the effects of climate change of relatively pristine systems^108^ such as old-growth forests^109^ has been overlooked in freshwater ecosystems^110^. It is plausible that late-successional forests reduce the consequences of thermal and hydrological stresses of climate change on the physiology and population dynamics of aquatic organisms. In Mack Creek, we show decreasing trends in the proportion of cold days (stream temperature ≤12 °C) consistent with steadily warming streams in winter over time^45^, potentially increasing temperature-size responses of aquatic organisms^17^. Forest management practices can be nuanced within and across streams^68–70^. In Needle Branch, forest management activities were much more detrimental to the stream channel and water quality in 1966 (clearcut with no buffer^65,66^) compared to 2009 (clearcut with buffer^64,72^). Potential increases in primary production and temperature (up to 30 °C)^66^ immediately after the clearcut in 1966 likely decreased when the second-growth canopy covered the stream. However, compared to pre-harvest conditions at Needle Branch, trout densities range from lower^111^ to relatively similar values^64,112^ over time. Collectively, our results illustrate the importance of considering legacy effects from past disturbance events in the face of climate change and their context-dependent associations to understand the importance of density-dependent and density-independent factors that influence body size of organisms.

### Further research to understand indirect climate effects on body size

The role of climate warming affecting food, habitat availability, and emigration, and the effect of their interacting factors on shrinking sizes of trout and salamanders is complex, but merits future consideration. To our knowledge, comprehensive time series of food or cover availability and patterns of emigration in headwater streams over time do not exist for trout or salamanders. It is plausible that stream-living trout and salamanders are not limited by food owing to the predicted global increase in secondary production^113^ and insects^114^ due to climate warming. Alternatively, large individuals might emigrate downstream to larger habitats when their metabolic demands and competition with conspecifics^54^ make downstream areas more conducive to growth and survival. Further, the increase in frequency and magnitude of droughts in a warming climate^115^ can negatively affect habitat size, diversity, and connectivity^116,117^ including warmer waters and low dissolved oxygen concentrations^116^. These events can also affect seasonal food availability^118,119^ and species interactions^117,120^ resulting in lower annual growth^50^ and thus, indirectly affect body size.

The ecological implications of smaller sizes in trout and stream salamanders owing to climate warming and their potential changes in life histories of species are difficult to predict given the multiple independent factors and mechanisms that could be involved. Smaller size can result in lower fitness and fecundity^4^, diminished swimming speed or power^121^, and competitive disadvantages within conspecifics^5^ and among species^6^. Climate warming can have both short-term (e.g., energy expenditures as YOY) and long-term (e.g., how energy allocations affect life-history expression later in life) effects that will be difficult to separate, and further experiment under controlled conditions are warranted. For example, investment in gonadal development, especially eggs in females, can affect realized growth as metabolic costs likely increase with warmer environments^122^. Prolonged exposure to warming stress during early life stages of trout can negatively impact reproduction, but offsprings of next generation receiving similar thermal stress have shown higher growth and survival, suggesting a potential rapid adaptation to climate warming^123^. These complex developmental and evolutionary aspects are modulated by both individual condition and the environment^124^. Here, we focused on single effects without interactions, but our findings are sufficient to illustrate the potential role of multiple factors regulating size. Nevertheless, changes in body size resulting from climate warming will likely have far-reaching effects from individuals to ecosystems^7^. More long-term efforts across ecosystems are urgently needed to generalize predictions about the ecological responses of observed declines in size. Ultimately, expected changes in ecosystems related to climate warming are complex and occur at multiple scales involving biological and physical processes related to species and their life histories.

## Conclusions

Our long-term data reveal compelling decreases in size of Coastal Cutthroat Trout, but not in Coastal Giant Salamanders. The associated mechanisms that explain these trends appear to be specific to local ecological and environmental contexts. Factors related to climate change, such as water temperature and flow variability, are related to the changes in size over time. However, size reductions also seem to be influenced by additional drivers unrelated to climate, such as density dependence. Detailed knowledge of the range of factors affecting size and the understanding of the complexity of species and ecosystems is crucial to effectively inform management and policy makers as they seek to adapt to climate change. Discoveries using these long-term datasets were not anticipated when these studies were designed, however, data on these species offer important insights allowing for the detection of environmental change, demonstrating the value of long-term ecological research.

### Supplementary Information

Supplementary Information 1.

Supplementary Information 2.

Supplementary Information 3.

## Data Availability

All data are available and archived at the following sites: 10.6073/pasta/7c78d662e847cdbe33584add8f809165, 10.6073/pasta/9437d1603044f5b92189110dd8343763, 10.6073/pasta/0066d6b04e736af5f234d95d97ee84f3.
