# Supplementary material for: Shrinking sizes of trout and salamanders are unexplained by climate warming alone
Source: Sci Rep. 2024 Jun 13;14:13614. doi: 10.1038/s41598-024-64145-x (PMC11176353; doi:10.1038/s41598-024-64145-x)
Supplement: Supplementary file 2 — Supplementary Information 2. [file 41598_2024_64145_MOESM2_ESM.docx]

**Supporting Information**

1. **Detailed animal sampling procedures**

At Mack Creek (old-growth and second-growth sections), we used datasets obtained by sampling freshwater vertebrates from the same three contiguous 50-m stream sections (150 m total) during the first week of September every year (Gregory & Arismendi, 2020). Trout data were available from 1987 through 2022; for salamanders data were available from 1993 through 2022. In 2020, Mack Creek was not sampled because the H. J. Andrews Experimental Forest was closed owing to a large wildfire, which occurred during the sampling period (Holiday Farm fire: 7 September–29 October 2020). Datasets were obtained using standard electrofishing procedures with block nets for each 50-m section. Abundance was estimated using two-pass depletion estimation (Seber & Le Cren, 1967) to reduce mortalities and minimize negative sampling impacts on the population. Probability of capture always exceeded 0.7 in all sections; two-sample depletion is unbiased if probability of capture exceeds 0.2 (Stewart et al., 2019). In our analysis, we distinguished adult trout (Age 1+; FL > 70 mm) from young-of-year trout (YOY; FL ≤ 70 mm) using a visual evaluation of breaks between length classes on length-frequency histograms (Fig. S1). For salamanders, however, we considered all size data owing to the difficulty of determining age based on length (Fig. S2).

For Flynn Creek and Needle Branch, we used data for stream-living adult Costal Cutthroat Trout (FL > 75; Fig. S1); Coastal Giant Salamanders were not sampled. Fish sampling was conducted in August and September. Because the Alsea Watershed Study was a multi-year effort by different research groups to estimate animal population sizes under different forest-management practices (Stednick, 2008), fish-capture protocols have differed over time. During the period 1962–1974, researchers used a mark-recapture protocol in stream sections ranging from 800 to 1500 m long (Moring, 1975). From 1988 through 1997, researchers used a 2-pass-removal standard electrofishing procedure with sampling stratified by section type that covered sections ranging from 260 to 300 m long (Gregory et al., 2008). In 2006–2017, researchers used single-pass electrofishing focusing on pools and cascades (Bateman et al., 2018) over sections of similar length to those from the period 1962–1974 (Moring, 1975). Some of the changes in protocols for animal capture might affect estimates of population abundance (Bateman et al., 2005; Foley et al, 2015; Peterson et al., 2004; Peterson & Cederholm, 1984; Thompson & Rahel, 1996), but will not affect patterns of body size in adults (Reid et al., 2008). In fact, electrofishing immobilization (Dolan & Miranda, 2003) and capture (Peterson et al., 2004) are more effective for larger animals. Potential sampling biases were minimized by maintaining consistent timing of sampling (i.e., low discharge and turbidity conditions occurred during August–September), study sections, and gear configuration for each site (Bonar et al., 2009; Breton et al., 2013).

1. **Model covariates in Mack Creek**

For Mack Creek, we used local available time series (Johnson et al., 2020; Gregory & Johnson, 2019; Gregory & Arismendi, 2020) of both density-dependent (i.e., proportion of YOY and abundance of freshwater vertebrates) and density-independent factors (i.e., temperature, discharge, and habitat size) to explore covariates that might affect size over time. We did not include density-dependent factors related to biomass because time series of body mass were discontinuous.

Specifically, as density-dependent factors we included the mean abundance of Coastal Cutthroat Trout YOY (#/50 m), mean abundance of Age 1+ Coastal Cutthroat Trout (#/50 m), and mean abundance of Coastal Giant Salamander (#/50 m) estimated from our annual sampling events (see above). For density-independent factors, we used annual/seasonal metrics of temperature (Arismendi et al., 2013) and discharge (Olden & Poff, 2003) that describe the seasonality of hydrological regimes (i.e., magnitude and variability) influenced by both snow and rain typical of our study region. For stream temperature, we included the annual proportion of cold days (daily mean ≤ 12°C), the annual proportion of optimal conditions for trout growth (daily mean >12°C and <15°C; peak growth of *O. clarkii* based on Bear et al. [2007]), winter (mean 7-days moving temperature DJF – daily min time series; °C) and summer (mean 7-days moving temperature JJA – daily max time series; °C) thermal conditions. For stream discharge, we included summer variability of stream discharge (CV in monthly discharge for JJA—daily min time series), stream discharge reversal (proportion of the year with negative changes in flow from one day to the next—daily mean time series), maximum stream discharge (mean annual 1-day discharge—daily max time series; m^3^/s), ratio between summer and annual stream discharge (mean 1-day summer JJA discharge divided by annual median discharge—daily min time series), and ratio between winter and annual stream discharge (mean 1-day winter DJF discharge divided by median discharge—daily max time series). Both temperature and stream discharge metrics were estimated using available daily time series from a gage station located in Mack Creek (Johnson et al., 2020; Gregory & Johnson, 2019). Daily time series were available for the entire study period except for 1989, 1991, 1993, 2010 and 2011 where data gaps in the time series did not allow for the estimation of all temperature metrics. In addition, we added local habitat-size information from our annual sampling events (Gregory & Arismendi, 2020) including the maximum depth of cascades, pools, and side channels within each section from Mack Creek.

1. **References**

Arismendi, I., Johnson, S. L., Dunham, J. B., & Haggerty, R. (2013). Descriptors of natural thermal regimes in streams and their responsiveness to change in the Pacific Northwest of North America. *Freshwater Biology, 58*(5), 880–894. doi:10.1111/fwb.12094

Bateman, D. S., Gresswell, R. E., & Torgersen, C. E. (2005). Evaluating single-pass catch as a tool for identifying spatial pattern in fish distribution. *Journal of Freshwater Ecology, 20*(2), 335–345. doi:10.1080/02705060.2005.9664974

Bateman, D. S., Gresswell, R. E., Warren, D., Hockman-Wert, D. P., Leer, D. W., Light, J. T., & Stednick, J. D. (2018). Fish response to contemporary timber harvest practices in a second-growth forest from the central Coast Range of Oregon. *Forest Ecology and Management, 411*, 142–157. doi:https://doi.org/10.1016/j.foreco.2018.01.030

Bear, E. A., McMahon, T. E., & Zale, A. V. (2007). Comparative thermal requirements of Westslope Cutthroat Trout and Rainbow Trout: implications for species interactions and development of thermal protection standards. *Transactions of the American Fisheries Society*, 136, 1113–1121. <https://doi.org/10.1577/T06-072.1>

Bonar, S. A., Hubert, W. A., & Willis, D. W. (2009). *Standard methods for sampling North American freshwater fishes*. American Fisheries Society.

Breton, A. R., Hawkins, J. A., & Winkelman, D. L. (2013). Correcting length–frequency distributions for imperfect detection. *North American Journal of Fisheries Management, 33*(6), 1156–1165. doi:10.1080/02755947.2013.829141

Dolan, C. R., & Miranda, L. E. (2003). Immobilization thresholds of electrofishing relative to fish size. *Transactions of the American Fisheries Society, 132*(5), 969–976. doi:10.1577/t02-055

Foley, K., Rosenberger, A., & Mueter, F. (2015). Effectiveness of single-pass backpack electrofishing to estimate juvenile coho salmon abundance in Alaskan headwater streams. *Fisheries Science, 81*(4), 601–610

Gregory, S., & Arismendi, I. (2020). Aquatic vertebrate population study in Mack Creek, Andrews Experimental Forest, 1987 to present. Long-Term Ecological Research. <https://doi.org/10.6073/pasta/7c78d662e847cdbe33584add8f809165>

Gregory, S., & Johnson, S. (2019). Stream and air temperature data from stream gages and stream confluences in the Andrews Experimental Forest, 1950 to present. Long-Term Ecological Research. <https://doi.org/10.6073/pasta/9437d1603044f5b92189110dd8343763.>

Gregory, S. V., Schwartz, J. S., Hall, J. D., Wildman, R. C., & Bisson, P. A. (2008). Long-term trends in habitat and fish populations in the Alsea basin. In Stednick, J. D., (Ed.), *Hydrological and biological responses to forest practices* (pp. 237–257). Springer.

Johnson, S., Wondzell, S., & Rothacher, J. (2020). Stream discharge in gaged watersheds at the HJ Andrews Experimental Forest, 1949 to present. Long-Term Ecological Research. <https://doi.org/10.6073/pasta/0066d6b04e736af5f234d95d97ee84f3.>

Moring, J. R. (1975). *The Alsea Watershed Study: effects of logging on the aquatic resources of three headwater streams of the Alsea River, Oregon Part I—biological studies*. Oregon State University. https://ir.library.oregonstate.edu/concern/technical_reports/765372384

Olden, J. D., & Poff, N. L. (2003). Redundancy and the choice of hydrologic indices for characterizing streamflow regimes. *River Research and Applications, 19*(2), 101–121. doi:10.1002/rra.700

Peterson, J. T., Thurow, R. F., & Guzevich, J. W. (2004). An evaluation of multipass electrofishing for estimating the abundance of stream‐dwelling salmonids. *Transactions of the American Fisheries Society, 133*(2), 462–475

Peterson, N., & Cederholm, C. (1984). A comparison of the removal and mark-recapture methods of population estimation for juvenile coho salmon in a small stream. *North American Journal of Fisheries Management, 4*(1), 99–102

Reid, S. M., Jones, N. E., & Yunker, G. (2008). Evaluation of single-pass electrofishing and rapid habitat assessment for monitoring Redside Dace. *North American Journal of Fisheries Management, 28*(1), 50–56. doi:10.1577/M06-283.1

Seber, G. A. F., & Le Cren, E. D. (1967). Estimating population parameters from catches large relative to the population. *Journal of Animal Ecology, 36*(3), 631–643. doi:10.2307/2818

Stednick, J. D. (Ed.) (2008). *Hydrological and biological responses to forest practices*. Springer.

Stewart, D. R., Butler, M. J., Johnson, L. A., Cajero, A., Young, A. N., & Harris, G. M. (2019). Efficacy of depletion models for estimating abundance of endangered fishes in streams. *Fisheries Research, 209*, 208–217. doi:https://doi.org/10.1016/j.fishres.2018.09.026

Thompson, P. D., & Rahel, F. J. (1996). Evaluation of depletion‐removal electrofishing of brook trout in small Rocky Mountain streams. *North American Journal of Fisheries Management, 16*(2), 332–339

1. **Supplementary Figures**

**Fig. S1.** Length-frequency histograms for Coastal Cutthroat Trout at the four study sites.

**
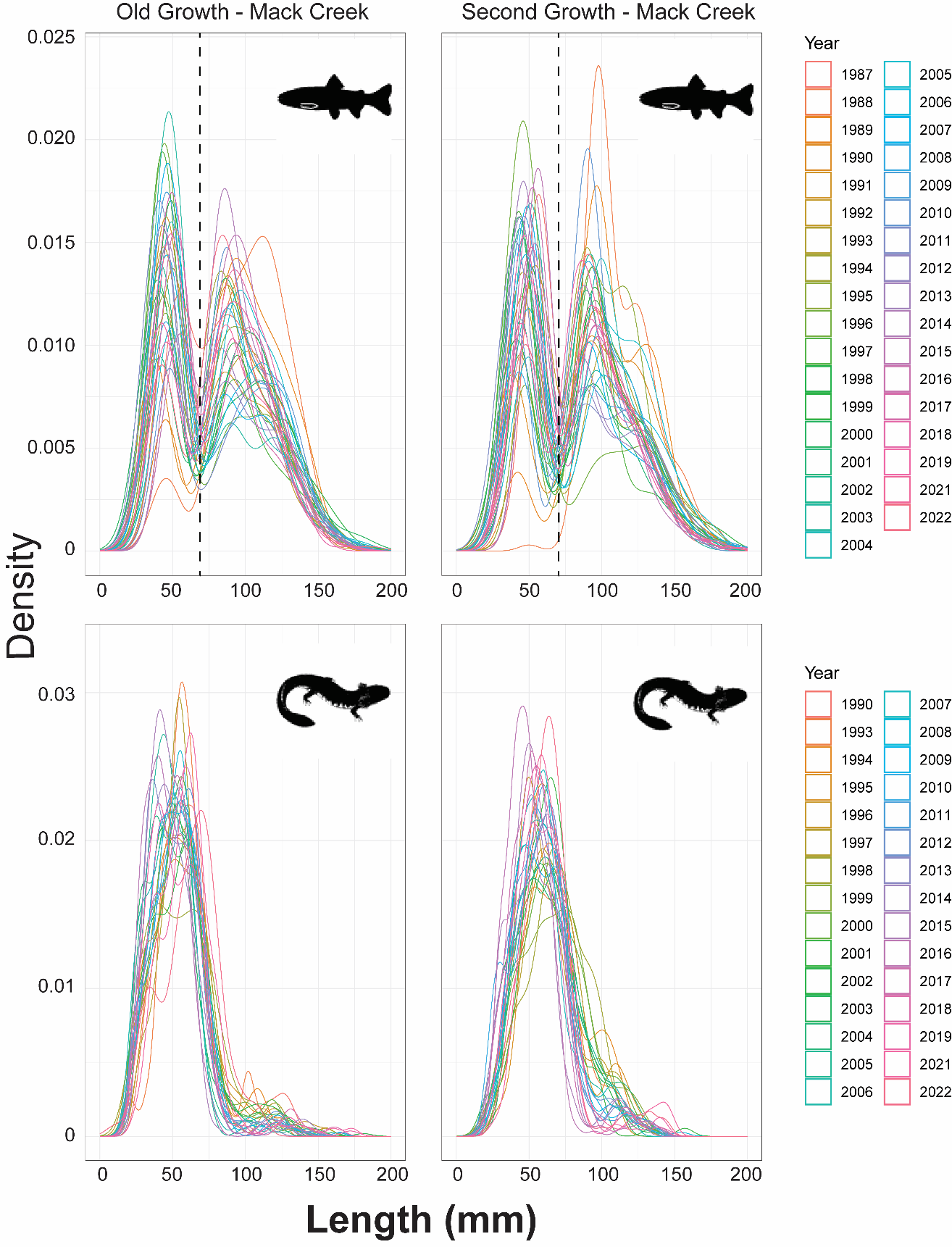
**

**Fig. S2.** Annual length-frequency histograms for Coastal Cutthroat Trout and Coastal Giant Salamander at Mack Creek study sites. The dotted line indicates separation between trout YOY and adult trout (FL = 70 mm).

1. **Supplementary Tables**

**Table S1.** Trends in size percentiles of Coastal Cutthroat Trout over the entire period of record analyzed using the Mann-Kendall test and Sen’s slope estimator (mm per decade) at Flynn Creek. Z value = Mann-Kendall Z statistic from original data; Sen’s slope = Sen’s slope from the original data; S = Mann-Kendall S statistic; Kendall’s Tau = Mann-Kendall’s Tau; BCP Z value = Bias corrected prewhitened Z value; BCP Sen’s slope = Bias corrected prewhitened Sen’s slope; BCP S = Bias corrected prewhitened S; BCP Kendall’sTau = Bias corrected prewhitened Kendall’s Tau; Bootstrapped *p*-value = Mann-Kendall bootstrapped *p*-value. Non-significant trends (ns), *p* < 0.10 (.), *p* < 0.05 (*), *p* < 0.01 (**), and *p* < 0.001 (***).

| **Percentile** | **Z value** | **Sen's slope** | **S** | ***p*-value** | **Kendall's Tau** | **BCP Z value** | **BCP Sen's slope** | **BCP S** | **BCP Kendall's Tau** | **Bootstrapped *p*-value** | **Statistical significance** |
| --- | --- | --- | --- | --- | --- | --- | --- | --- | --- | --- | --- |
| 5 | -1.30 | -0.83 | -88 | 0.194 | -0.16 | -0.36 | -0.32 | -24 | -0.05 | 0.365 | ns |
| 10 | -2.05 | -1.11 | -138 | 0.041 | -0.25 | -1.86 | -0.96 | -121 | -0.23 | **0.031** | * |
| 15 | -2.49 | -1.60 | -168 | 0.013 | -0.30 | -2.36 | -1.53 | -153 | -0.29 | **0.011** | * |
| 20 | -2.67 | -2.00 | -180 | 0.008 | -0.32 | -3.02 | -2.13 | -196 | -0.37 | **0.001** | ** |
| 25 | -2.81 | -2.07 | -189 | 0.005 | -0.34 | -3.01 | -1.99 | -195 | -0.37 | **0.002** | ** |
| 30 | -2.97 | -2.50 | -200 | 0.003 | -0.36 | -3.30 | -2.44 | -214 | -0.41 | **0.001** | *** |
| 35 | -3.27 | -2.59 | -220 | 0.001 | -0.39 | -3.74 | -2.86 | -242 | -0.46 | **0.001** | *** |
| 40 | -3.45 | -2.94 | -233 | 0.001 | -0.42 | -3.41 | -2.88 | -221 | -0.42 | **0.001** | *** |
| 45 | -3.55 | -2.86 | -239 | 0.000 | -0.43 | -3.72 | -2.97 | -241 | -0.46 | **0.001** | *** |
| 50 | -3.47 | -3.16 | -234 | 0.001 | -0.42 | -3.72 | -3.17 | -241 | -0.46 | **0.001** | *** |
| 55 | -4.13 | -3.81 | -278 | 0.000 | -0.50 | -4.35 | -4.45 | -282 | -0.53 | **0.001** | *** |
| 60 | -4.74 | -4.71 | -319 | 0.000 | -0.57 | -4.83 | -5.18 | -313 | -0.59 | **0.001** | *** |
| 65 | -4.57 | -5.00 | -308 | 0.000 | -0.55 | -4.83 | -5.78 | -313 | -0.59 | **0.001** | *** |
| 70 | -4.66 | -5.63 | -314 | 0.000 | -0.56 | -5.10 | -6.65 | -330 | -0.63 | **0.001** | *** |
| 75 | -4.75 | -6.96 | -320 | 0.000 | -0.57 | -4.77 | -7.09 | -309 | -0.59 | **0.001** | *** |
| 80 | -5.26 | -8.00 | -355 | 0.000 | -0.63 | -4.85 | -7.49 | -314 | -0.59 | **0.001** | *** |
| 85 | -5.48 | -10.00 | -370 | 0.000 | -0.66 | -5.07 | -9.38 | -328 | -0.62 | **0.001** | *** |
| 90 | -5.57 | -12.22 | -376 | 0.000 | -0.67 | -5.41 | -11.36 | -350 | -0.66 | **0.001** | *** |
| 95 | -5.55 | -14.00 | -375 | 0.000 | -0.67 | -4.23 | -7.80 | -274 | -0.52 | **0.001** | *** |

**Table S2.** Trends in size percentiles of Coastal Cutthroat Trout over the entire period of record analyzed using the Mann-Kendall test and Sen’s slope estimator (mm per decade) at Needle Branch. Z value = Mann-Kendall Z statistic from original data; Sen’s slope = Sen’s slope from the original data; S = Mann-Kendall S statistic; Kendall’s Tau = Mann-Kendall’s Tau; BCP Z value = Bias corrected prewhitened Z value; BCP Sen’s slope = Bias corrected prewhitened Sen’s slope; BCP S = Bias corrected prewhitened S; BCP Kendall’sTau = Bias corrected prewhitened Kendall’s Tau; Bootstrapped *p*-value = Mann-Kendall bootstrapped *p*-value. Non-significant trends (ns), *p* < 0.10 (·), *p* < 0.05 (*), *p* < 0.01 (**), and *p* < 0.001 (***).

| **Percentile** | **Z value** | **Sen's slope** | **S** | ***p*-value** | **Kendall's Tau** | **BCP Z value** | **BCP Sen's slope** | **BCP S** | **BCP Kendall's Tau** | **Bootstrapped *p*-value** | **Statistical significance** |
| --- | --- | --- | --- | --- | --- | --- | --- | --- | --- | --- | --- |
| 5 | -1.06 | -2.32 | -66 | 0.291 | -0.13 | -0.27 | -0.84 | -17 | -0.04 | 0.379 | ns |
| 10 | -1.02 | -2.95 | -64 | 0.306 | -0.13 | -0.71 | -1.62 | -43 | -0.09 | 0.236 | ns |
| 15 | -1.30 | -3.89 | -81 | 0.194 | -0.16 | -0.51 | -1.48 | -31 | -0.07 | 0.301 | ns |
| 20 | -1.46 | -4.40 | -91 | 0.144 | -0.18 | -0.71 | -2.00 | -43 | -0.09 | 0.227 | ns |
| 25 | -1.61 | -4.91 | -100 | 0.108 | -0.20 | -0.75 | -2.78 | -45 | -0.10 | 0.239 | ns |
| 30 | -1.65 | -5.00 | -103 | 0.098 | -0.21 | -0.99 | -2.64 | -59 | -0.13 | 0.143 | ns |
| 35 | -1.96 | -5.56 | -122 | 0.049 | -0.25 | -1.16 | -2.95 | -69 | -0.15 | 0.121 | ns |
| 40 | -2.03 | -5.88 | -126 | 0.042 | -0.25 | -1.22 | -3.71 | -73 | -0.16 | 0.105 | ns |
| 45 | -2.01 | -6.36 | -125 | 0.044 | -0.25 | -1.43 | -4.36 | -85 | -0.18 | 0.073 | · |
| 50 | -2.31 | -6.94 | -143 | 0.021 | -0.29 | -1.80 | -5.03 | -107 | -0.23 | **0.033** | * |
| 55 | -2.31 | -7.61 | -143 | 0.021 | -0.29 | -1.89 | -5.58 | -112 | -0.24 | **0.029** | * |
| 60 | -2.37 | -7.39 | -147 | 0.018 | -0.30 | -1.94 | -5.61 | -115 | -0.25 | **0.025** | * |
| 65 | -2.39 | -7.39 | -148 | 0.017 | -0.30 | -2.02 | -5.18 | -120 | -0.26 | **0.018** | * |
| 70 | -2.26 | -7.21 | -140 | 0.024 | -0.28 | -1.70 | -5.16 | -101 | -0.22 | **0.045** | * |
| 75 | -2.50 | -7.10 | -155 | 0.012 | -0.31 | -1.70 | -5.08 | -101 | -0.22 | **0.034** | * |
| 80 | -2.61 | -8.05 | -162 | 0.009 | -0.33 | -1.70 | -5.90 | -101 | -0.22 | **0.045** | * |
| 85 | -2.65 | -8.17 | -164 | 0.008 | -0.33 | -1.53 | -5.68 | -91 | -0.20 | 0.064 | .· |
| 90 | -2.99 | -9.80 | -185 | 0.003 | -0.37 | -1.63 | -5.81 | -97 | -0.21 | **0.044** | * |
| 95 | -3.25 | -11.40 | -201 | 0.001 | -0.41 | -2.55 | -8.65 | -151 | -0.32 | **0.005** | ** |

**Table S3.** Trends in size percentiles of Coastal Cutthroat Trout over the entire period of record analyzed using the Mann-Kendall test and Sen’s slope estimator (mm per decade) at the old-growth section of Mack Creek. Z value = Mann-Kendall Z statistic from original data; Sen’s slope = Sen’s slope from the original data; S = Mann-Kendall S statistic; Kendall’s Tau = Mann-Kendall’s Tau; BCP Z value = Bias corrected prewhitened Z value; BCP Sen’s Slope = Bias corrected prewhitened Sen’s slope; BCP S = Bias corrected prewhitened S; BCP Kendall’sTau = Bias corrected prewhitened Kendall’s Tau; Bootstrapped *p*-value = Mann-Kendall bootstrapped *p*-value. Non-significant trends (ns), *p* < 0.10 (·), *p* < 0.05 (*), *p* < 0.01 (**), and *p* < 0.001 (***).

| **Percentile** | **Z value** | **Sen's slope** | **S** | ***p*-value** | **Kendall's Tau** | **BCP Z value** | **BCP Sen's slope** | **BCP S** | **BCP Kendall's Tau** | **Bootstrapped *p*-value** | **Statistical significance** |
| --- | --- | --- | --- | --- | --- | --- | --- | --- | --- | --- | --- |
| 5 | -0.52 | 0.00 | -37 | 0.605 | -0.06 | -1.08 | -0.41 | -74 | -0.13 | 0.131 | ns |
| 10 | -0.77 | -0.43 | -55 | 0.440 | -0.09 | -1.26 | -0.67 | -86 | -0.15 | 0.097 | · |
| 15 | -0.82 | -0.34 | -58 | 0.414 | -0.10 | -1.38 | -0.84 | -94 | -0.17 | 0.072 | · |
| 20 | -0.86 | -0.48 | -61 | 0.390 | -0.10 | -1.11 | -0.75 | -76 | -0.14 | 0.133 | ns |
| 25 | -0.71 | -0.37 | -51 | 0.475 | -0.09 | -0.95 | -0.78 | -65 | -0.12 | 0.163 | ns |
| 30 | -0.94 | -0.71 | -67 | 0.346 | -0.11 | -1.32 | -1.05 | -90 | -0.16 | 0.098 | · |
| 35 | -0.96 | -0.87 | -68 | 0.339 | -0.11 | -1.22 | -1.35 | -83 | -0.15 | 0.098 | · |
| 40 | -0.95 | -0.91 | -68 | 0.340 | -0.11 | -1.20 | -1.29 | -82 | -0.15 | 0.118 | ns |
| 45 | -1.57 | -1.67 | -111 | 0.117 | -0.19 | -1.72 | -1.98 | -117 | -0.21 | **0.046** | * |
| 50 | -1.63 | -1.61 | -115 | 0.104 | -0.19 | -1.94 | -1.92 | -132 | -0.24 | **0.024** | * |
| 55 | -1.39 | -1.18 | -98 | 0.166 | -0.16 | -1.56 | -1.63 | -106 | -0.19 | **0.048** | * |
| 60 | -1.41 | -1.30 | -100 | 0.158 | -0.17 | -1.51 | -1.68 | -103 | -0.18 | 0.062 | · |
| 65 | -1.40 | -1.18 | -99 | 0.162 | -0.17 | -1.66 | -1.43 | -113 | -0.20 | **0.048** | * |
| 70 | -1.80 | -1.67 | -127 | 0.073 | -0.21 | -1.73 | -1.43 | -118 | -0.21 | **0.039** | * |
| 75 | -1.72 | -1.18 | -121 | 0.086 | -0.20 | -1.45 | -1.19 | -99 | -0.18 | 0.066 | · |
| 80 | -1.53 | -1.25 | -108 | 0.126 | -0.18 | -1.32 | -0.98 | -90 | -0.16 | 0.091 | · |
| 85 | -1.81 | -1.43 | -128 | 0.070 | -0.22 | -1.87 | -1.39 | -127 | -0.23 | **0.024** | * |
| 90 | -2.66 | -2.00 | -187 | 0.008 | -0.31 | -1.66 | -1.11 | -113 | -0.20 | **0.044** | * |
| 95 | -1.91 | -2.00 | -135 | 0.056 | -0.23 | -1.19 | -0.94 | -81 | -0.14 | 0.120 | · |

**Table S4.** Trends in size percentiles of Coastal Cutthroat Trout over the entire period of record analyzed using the Mann-Kendall test and Sen’s slope estimator (mm per decade) at the second-growth section of Mack Creek. Z value = Mann-Kendall Z statistic from original data; Sen’s slope = Sen’s slope from the original data; S = Mann-Kendall S statistic; Kendall’s Tau = Mann-Kendall’s Tau; BCP Z value = Bias corrected prewhitened Z value; BCP Sen’s slope = Bias corrected prewhitened Sen’s slope; BCP S = Bias corrected prewhitened S; BCP Kendall’sTau = Bias corrected prewhitened Kendall’s Tau; Bootstrapped *p*-value = Mann-Kendall bootstrapped *p*-value. Non-significant trends (ns), *p* < 0.10 (·), *p* < 0.05 (*), *p* < 0.01 (**), and *p* < 0.001 (***).

| **Percentile** | **Z value** | **Sen's slope** | **S** | ***p*-value** | **Kendall's Tau** | **BCP Z value** | **BCP Sen's slope** | **BCP S** | **BCP Kendall's Tau** | **Bootstrapped *p*-value** | **Statistical significance** |
| --- | --- | --- | --- | --- | --- | --- | --- | --- | --- | --- | --- |
| 5 | -0.80 | -0.56 | -57 | 0.424 | -0.10 | -0.73 | -0.52 | -50 | -0.09 | 0.220 | ns |
| 10 | -1.61 | -1.00 | -114 | 0.107 | -0.19 | -1.05 | -0.82 | -72 | -0.13 | 0.139 | ns |
| 15 | -2.20 | -1.43 | -155 | 0.028 | -0.26 | -1.16 | -0.94 | -79 | -0.14 | 0.110 | ns |
| 20 | -2.68 | -1.82 | -189 | 0.007 | -0.32 | -1.69 | -1.34 | -115 | -0.20 | **0.046** | * |
| 25 | -2.53 | -1.82 | -178 | 0.011 | -0.30 | -1.51 | -1.30 | -103 | -0.18 | 0.064 | · |
| 30 | -2.87 | -1.88 | -202 | 0.004 | -0.34 | -1.99 | -1.45 | -135 | -0.24 | **0.029** | * |
| 35 | -2.40 | -1.67 | -169 | 0.017 | -0.28 | -1.94 | -1.67 | -132 | -0.24 | **0.026** | * |
| 40 | -2.14 | -1.43 | -150 | 0.033 | -0.25 | -1.90 | -1.51 | -129 | -0.23 | **0.025** | * |
| 45 | -1.78 | -1.67 | -126 | 0.074 | -0.21 | -1.72 | -1.69 | -117 | -0.21 | **0.032** | * |
| 50 | -1.78 | -1.88 | -126 | 0.075 | -0.21 | -1.69 | -2.16 | -115 | -0.20 | **0.044** | * |
| 55 | -1.80 | -2.00 | -127 | 0.072 | -0.21 | -1.82 | -2.31 | -124 | -0.22 | **0.034** | * |
| 60 | -1.83 | -2.22 | -129 | 0.068 | -0.22 | -1.90 | -2.64 | -129 | -0.23 | **0.027** | * |
| 65 | -1.77 | -2.00 | -125 | 0.077 | -0.21 | -2.03 | -2.39 | -138 | -0.25 | **0.013** | * |
| 70 | -1.98 | -2.07 | -140 | 0.047 | -0.24 | -2.30 | -2.47 | -156 | -0.28 | **0.008** | ** |
| 75 | -1.88 | -2.00 | -133 | 0.060 | -0.22 | -2.09 | -2.30 | -142 | -0.25 | **0.015** | * |
| 80 | -2.37 | -2.27 | -167 | 0.018 | -0.28 | -2.61 | -2.34 | -177 | -0.32 | **0.005** | ** |
| 85 | -2.31 | -2.14 | -163 | 0.021 | -0.27 | -2.27 | -1.98 | -154 | -0.27 | **0.011** | ** |
| 90 | -3.28 | -2.50 | -231 | 0.001 | -0.39 | -2.85 | -2.25 | -193 | -0.34 | **0.001** | *** |
| 95 | -2.84 | -2.94 | -200 | 0.005 | -0.34 | -3.23 | -3.18 | -219 | -0.39 | **0.001** | *** |

**Table S5.** Trends in size percentiles of Coastal Giant Salamander over the entire period of record analyzed using the Mann-Kendall test and Sen’s slope estimator (mm per decade) at the old-growth section of Mack Creek. Z value = Mann-Kendall Z statistic from original data; Sen’s slope = Sen’s slope from the original data; S = Mann-Kendall S statistic; Kendall’s Tau = Mann-Kendall’s Tau; BCP Z value = Bias corrected prewhitened Z value; BCP Sen’s slope = Bias corrected prewhitened Sen’s slope; BCP S = Bias corrected prewhitened S; BCP Kendall’sTau = Bias corrected prewhitened Kendall’s Tau; Bootstrapped *p*-value = Mann-Kendall bootstrapped *p*-value. Non-significant trends (ns), *p* < 0.10 (·), *p* < 0.05 (*), *p* < 0.01 (**), and *p* < 0.001 (***).

| **Percentile** | **Z Value** | **Sen's Slope** | **S** | ***p*-value** | **Kendall's Tau** | **BCP Z Value** | **BCP Sen's Slope** | **BCP S** | **BCP Kendall's Tau** | **Bootstrapped *p*-value** | **Statistical significance** |
| --- | --- | --- | --- | --- | --- | --- | --- | --- | --- | --- | --- |
| 5 | 0.00 | 0.00 | 0 | 1.000 | 0.00 | 0.65 | 0.47 | 34 | 0.09 | 0.738 | ns |
| 10 | -0.38 | 0.00 | -21 | 0.705 | -0.05 | 0.00 | 0.01 | 1 | 0.00 | 0.521 | ns |
| 15 | -1.28 | -0.80 | -68 | 0.200 | -0.17 | -0.53 | -0.26 | -28 | -0.07 | 0.287 | ns |
| 20 | -1.77 | -1.50 | -95 | 0.076 | -0.23 | -0.57 | -0.52 | -30 | -0.08 | 0.290 | ns |
| 25 | -1.92 | -2.00 | -103 | 0.055 | -0.25 | 0.16 | 0.06 | 9 | 0.02 | 0.561 | ns |
| 30 | -1.55 | -1.58 | -83 | 0.121 | -0.20 | 0.14 | 0.28 | 8 | 0.02 | 0.550 | ns |
| 35 | -1.57 | -1.79 | -84 | 0.117 | -0.21 | 0.22 | 0.15 | 12 | 0.03 | 0.580 | ns |
| 40 | -1.64 | -1.72 | -88 | 0.100 | -0.22 | -0.02 | -0.06 | -2 | -0.01 | 0.474 | ns |
| 45 | -2.12 | -2.14 | -113 | 0.034 | -0.28 | -0.02 | 0.00 | -2 | -0.01 | 0.496 | ns |
| 50 | -1.98 | -2.00 | -106 | 0.047 | -0.26 | 0.30 | 0.49 | 16 | 0.04 | 0.616 | ns |
| 55 | -1.83 | -1.85 | -98 | 0.067 | -0.24 | 1.28 | 1.23 | 66 | 0.17 | 0.913 | ns |
| 60 | -2.02 | -2.12 | -108 | 0.044 | -0.27 | 1.56 | 1.57 | 80 | 0.21 | 0.935 | ns |
| 65 | -1.93 | -1.70 | -103 | 0.054 | -0.25 | 2.02 | 1.71 | 103 | 0.27 | 0.981 | ns |
| 70 | -1.85 | -2.00 | -99 | 0.064 | -0.24 | 1.60 | 1.33 | 82 | 0.22 | 0.943 | ns |
| 75 | -1.97 | -2.22 | -105 | 0.049 | -0.26 | 1.11 | 0.82 | 57 | 0.15 | 0.877 | ns |
| 80 | -2.08 | -2.00 | -111 | 0.038 | -0.27 | 1.11 | 0.91 | 57 | 0.15 | 0.867 | ns |
| 85 | -2.16 | -2.50 | -116 | 0.030 | -0.29 | 1.72 | 1.27 | 88 | 0.23 | 0.959 | ns |
| 90 | -2.92 | -5.00 | -156 | 0.003 | -0.38 | 0.47 | 0.56 | 25 | 0.07 | 0.686 | ns |
| 95 | -3.53 | -11.58 | -189 | 0.000 | -0.47 | 0.10 | 0.35 | 6 | 0.02 | 0.560 | ns |

**Table S6.** Trends in size percentiles of Coastal Giant Salamander over the entire period of record analyzed using the Mann-Kendall test and Sen’s slope estimator (mm per decade) at the second-growth section of Mack Creek. Z value = Mann-Kendall Z statistic from original data; Sen’s slope = Sen’s slope from the original data; S = Mann-Kendall S statistic; Kendall’s Tau = Mann-Kendall’s Tau; BCP Z value = Bias corrected prewhitened Z value; BCP Sen’s slope = Bias corrected prewhitened Sen’s slope; BCP S = Bias corrected prewhitened S; BCP Kendall’sTau = Bias corrected prewhitened Kendall’s Tau; Bootstrapped *p*-value = Mann-Kendall bootstrapped *p*-value. Non-significant trends (ns), *p* < 0.10 (·), *p* < 0.05 (*), *p* < 0.01 (**), and *p* < 0.001 (***).

| **Percentile** | **Z value** | **Sen's slope** | **S** | ***p*-value** | **Kendall's Tau** | **BCP Z value** | **BCP Sen's slope** | **BCP S** | **BCP Kendall's Tau** | **Bootstrapped *p*-value** | **Statistical significance** |
| --- | --- | --- | --- | --- | --- | --- | --- | --- | --- | --- | --- |
| 5 | -1.59 | -1.56 | -85 | 0.113 | -0.21 | -1.52 | -1.60 | -78 | -0.21 | 0.058 | · |
| 10 | -2.66 | -2.00 | -141 | 0.008 | -0.35 | -1.27 | -0.86 | -65 | -0.17 | 0.105 | ns |
| 15 | -2.09 | -2.11 | -112 | 0.036 | -0.28 | -1.40 | -1.34 | -72 | -0.19 | 0.089 | · |
| 20 | -2.38 | -2.50 | -127 | 0.017 | -0.31 | -1.46 | -1.95 | -75 | -0.20 | 0.072 | · |
| 25 | -3.41 | -3.51 | -182 | 0.001 | -0.45 | -0.71 | -0.62 | -37 | -0.10 | 0.244 | ns |
| 30 | -3.37 | -3.33 | -179 | 0.001 | -0.44 | -0.91 | -0.82 | -47 | -0.12 | 0.173 | ns |
| 35 | -3.01 | -2.82 | -160 | 0.003 | -0.39 | -0.81 | -0.97 | -42 | -0.11 | 0.207 | ns |
| 40 | -3.07 | -3.19 | -164 | 0.002 | -0.40 | -0.99 | -0.96 | -51 | -0.13 | 0.161 | ns |
| 45 | -3.22 | -3.33 | -172 | 0.001 | -0.42 | -1.09 | -1.20 | -56 | -0.15 | 0.116 | ns |
| 50 | -3.65 | -3.51 | -194 | 0.000 | -0.48 | -1.15 | -1.12 | -59 | -0.16 | 0.113 | ns |
| 55 | -3.81 | -4.29 | -203 | 0.000 | -0.50 | -0.91 | -1.39 | -47 | -0.12 | 0.172 | ns |
| 60 | -3.71 | -4.17 | -198 | 0.000 | -0.49 | -0.85 | -0.65 | -44 | -0.12 | 0.189 | ns |
| 65 | -3.82 | -4.25 | -204 | 0.000 | -0.50 | -1.03 | -1.11 | -53 | -0.14 | 0.153 | ns |
| 70 | -3.96 | -4.44 | -211 | 0.000 | -0.52 | -1.54 | -1.43 | -79 | -0.21 | 0.059 | · |
| 75 | -3.97 | -5.00 | -212 | 0.000 | -0.52 | -1.48 | -1.76 | -76 | -0.20 | 0.067 | · |
| 80 | -4.13 | -6.00 | -220 | 0.000 | -0.54 | -2.37 | -3.54 | -121 | -0.32 | **0.006** | ** |
| 85 | -4.39 | -6.67 | -234 | 0.000 | -0.58 | -2.98 | -4.40 | -152 | -0.40 | **0.001** | *** |
| 90 | -4.62 | -10.00 | -247 | 0.000 | -0.61 | -3.77 | -7.13 | -192 | -0.51 | **0.001** | *** |
| 95 | -1.60 | -3.88 | -86 | 0.110 | -0.21 | -1.42 | -3.01 | -73 | -0.19 | 0.067 | · |

**Table S7** Sample size for body size (mm) of Coastal Cutthroat Trout (Fork Length; FL) and Coastal Giant Salamander (Snout-to-vent; SVL) by year at Mach Creek old-growth and second-growth sections (H.J. Andrews Experimental Forest).

|  | **Coastal Cutthroat Trout adults (>70 mm FL)** | | **Coastal Giant Salamander** | |
| --- | --- | --- | --- | --- |
| **Year** | **Second-growth section** | **Old-growth section** | **Second-growth section** | **Old-growth section** |
| 1987 | 224 | 150 |  |  |
| 1988 | 192 | 99 |  |  |
| 1989 | 172 | 99 |  |  |
| 1990 | 212 | 164 |  |  |
| 1991 | 228 | 134 |  |  |
| 1992 | 238 | 149 |  |  |
| 1993 | 176 | 170 | 126 | 129 |
| 1994 | 270 | 180 | 169 | 137 |
| 1995 | 145 | 139 | 63 | 130 |
| 1996 | 117 | 151 | 90 | 147 |
| 1997 | 268 | 175 | 97 | 132 |
| 1998 | 272 | 233 | 77 | 159 |
| 1999 | 195 | 166 | 80 | 152 |
| 2000 | 201 | 162 | 170 | 210 |
| 2001 | 238 | 199 | 133 | 190 |
| 2002 | 219 | 132 | 266 | 384 |
| 2003 | 121 | 119 | 360 | 372 |
| 2004 | 176 | 163 | 192 | 251 |
| 2005 | 181 | 198 | 260 | 309 |
| 2006 | 161 | 180 | 178 | 246 |
| 2007 | 202 | 168 | 252 | 305 |
| 2008 | 249 | 229 | 258 | 373 |
| 2009 | 223 | 200 | 261 | 348 |
| 2010 | 229 | 204 | 264 | 308 |
| 2011 | 221 | 175 | 328 | 371 |
| 2012 | 229 | 188 | 231 | 224 |
| 2013 | 209 | 174 | 184 | 227 |
| 2014 | 227 | 250 | 137 | 143 |
| 2015 | 178 | 174 | 245 | 181 |
| 2016 | 146 | 126 | 264 | 202 |
| 2017 | 167 | 146 | 368 | 328 |
| 2018 | 198 | 146 | 171 | 212 |
| 2019 | 191 | 142 | 184 | 189 |
| 2020^1^ | n.a. | n.a. | n.a. | n.a. |
| 2021 | 179 | 151 | 173 | 160 |
| 2022 | 205 | 153 | 162 | 100 |

^1^No sampling due to the Holiday Farm Fire that occurred during the sampling season.

**Table S8** Sample size for body size (mm) of Coastal Cutthroat Trout (Fork Length; FL) by year at Flynn Creek and Needle Branch (Alsea Watershed Study sites).

|  | **Coastal Cutthroat Trout adults**  **(> 75 mm FL)** | |
| --- | --- | --- |
| **Year** | **Flynn Creek** | **Needle Branch** |
| 1962 | 200 | 41 |
| 1963 | 207 | 158 |
| 1964 | 441 | 206 |
| 1965 | 341 | 117 |
| 1966 | 477 | 59 |
| 1967 | 675 | 13 |
| 1968 | 664 | 72 |
| 1969 | 1087 | 209 |
| 1970 |  | 54 |
| 1971 | 632 | 57 |
| 1972 | 827 | 65 |
| 1973 | 462 | 46 |
| 1974 | 606 | 178 |
| 1988 | 132 |  |
| 1989 | 97 | 15 |
| 1990 | 63 | 23 |
| 1991 | 141 | 25 |
| 1992 | 91 |  |
| 1993 | 90 | 21 |
| 1994 | 75 | 12 |
| 1995 | 80 | 16 |
| 1996 | 59 | 26 |
| 1997 | 84 |  |
| 2006 | 345 | 140 |
| 2007 | 253 | 42 |
| 2008 | 293 | 146 |
| 2009 | 249 | 152 |
| 2010 | 239 | 234 |
| 2011 | 202 | 184 |
| 2012 | 180 | 318 |
| 2013 | 372 | 262 |
| 2014 | 359 | 316 |
| 2015 | 276 | 110 |
| 2016 | 255 | 105 |
| 2017 | 231 | 190 |

**Table S9.** Trends in covariates over the entire period of record analyzed using the Mann-Kendall test and Sen’s slope estimator (unit per year) at both the old-growth (OG) and second-growth (SG) sections of Mack Creek (MC). See details about covariates in Table 1. Z value = Mann-Kendall Z statistic from original data; Sen’s slope = Sen’s slope from the original data; S = Mann-Kendall S statistic; Kendall’s Tau = Mann-Kendall’s Tau; BCP Z value = Bias corrected prewhitened Z value; BCP Sen’s slope = Bias corrected prewhitened Sen’s slope; BCP S = Bias corrected prewhitened S; BCP Kendall’sTau = Bias corrected prewhitened Kendall’s Tau; Bootstrapped *p*-value = Mann-Kendall bootstrapped *p*-value. Non-significant trends (ns), *p* < 0.10 (·), *p* < 0.05 (*), *p* < 0.01 (**), and *p* < 0.001 (***).

| **Covariate** | **Z value** | **Sen's slope** | **S** | ***p*-value** | **Kendall's Tau** | **BCP Z value** | **BCP Sen's slope** | **BCP S** | **BCP Kendall's Tau** | **Bootstrapped *p*-value** | **Statistical significance** |
| --- | --- | --- | --- | --- | --- | --- | --- | --- | --- | --- | --- |
| YOY^OG^ | 0.17 | 0.092 | 13 | 0.865 | 0.022 | -0.15 | -0.027 | -11 | -0.02 | 0.434 | ns |
| Trout_ab^OG^ | 0.98 | 0.233 | 70 | 0.327 | 0.118 | 0.33 | 0.068 | 23 | 0.04 | 0.641 | ns |
| Salamander_ab^OG^ | 0.88 | 0.855 | 48 | 0.378 | 0.118 | -0.61 | -0.526 | -32 | -0.08 | 0.269 | ns |
| Hab_size_cascade^OG^ | -1.62 | -0.002 | -87 | 0.106 | -0.214 | 0.00 | 0.000 | 0 | 0.00 | 0.503 | ns |
| Hab_size_pool^OG^ | 0.36 | 0.000 | 20 | 0.721 | 0.049 | -0.63 | -0.001 | -33 | -0.09 | 0.249 | ns |
| Hab_side_channel^OG^ | 0.00 | 0.000 | -1 | 1.000 | -0.002 | -1.05 | -0.001 | -54 | -0.14 | 0.146 | ns |
| YOY^SG^ | 0.57 | 0.208 | 41 | 0.570 | 0.069 | 0.77 | 0.271 | 53 | 0.09 | 0.779 | ns |
| Trout_ab^SG^ | -1.46 | -0.272 | -104 | 0.143 | -0.175 | -0.83 | -0.234 | -57 | -0.10 | 0.204 | ns |
| Salamander_ab^SG^ | 1.82 | 1.791 | 98 | 0.069 | 0.241 | 1.88 | 1.642 | 96 | 0.25 | 0.974 | ns |
| Hab_size_cascade^SG^ | -0.13 | 0.000 | -8 | 0.895 | -0.020 | -0.45 | 0.000 | -24 | -0.06 | 0.305 | ns |
| Hab_size_pool^SG^ | 1.60 | 0.004 | 86 | 0.110 | 0.212 | 0.95 | 0.001 | 49 | 0.13 | 0.821 | ns |
| Hab_side_channel^SG^ | -2.51 | -0.002 | -134 | 0.012 | -0.330 | -0.89 | -0.001 | -46 | -0.12 | 0.184 | ns |
| T_cold_events^MC^ | -2.63 | -0.002 | -154 | 0.008 | -0.331 | -1.86 | -0.002 | -105 | -0.24 | **0.033** | * |
| T_optima_events^MC^ | 2.63 | 0.002 | 154 | 0.008 | 0.331 | 1.86 | 0.002 | 105 | 0.24 | 0.962 | ns |
| T_winter^MC^ | 0.43 | 0.004 | 26 | 0.669 | 0.056 | 1.00 | 0.011 | 57 | 0.13 | 0.833 | ns |
| T_summer^MC^ | 3.00 | 0.053 | 169 | 0.003 | 0.389 | 2.49 | 0.048 | 134 | 0.33 | 0.995 | ns |
| Q_var_low^MC^ | -1.07 | -0.003 | -79 | 0.287 | -0.125 | -1.66 | -0.003 | -118 | -0.20 | **0.044** | * |
| Q_reversal^MC^ | 2.90 | 0.001 | 212 | 0.004 | 0.337 | 2.93 | 0.001 | 207 | 0.35 | 0.999 | ns |
| Q_max^MC^ | 1.06 | 0.037 | 79 | 0.288 | 0.125 | 0.88 | 0.032 | 63 | 0.11 | 0.819 | ns |
| Q_ratio_min^MC^ | -0.31 | -0.001 | -24 | 0.754 | -0.038 | -0.43 | -0.001 | -31 | -0.05 | 0.329 | ns |
| Q_ratio_max^MC^ | 1.16 | 0.024 | 86 | 0.247 | 0.137 | 0.94 | 0.022 | 67 | 0.11 | 0.835 | ns |

**Table S10** Top-supported models (Akaike information criterion corrected for small sample sizes AICc difference among models < 2) and their performance relating body size (median length) of Coastal Cutthroat Trout and both biotic and abiotic factors (f) in Mack Creek, Oregon Cascades. There were 16,950 candidate models (2^f^) tested after correcting for multicollinearity and missing data. Best-supported models included both density-dependent (abundance of animals) and density-independent (Q = flow; T = temperature; H = habitat size) factors. Biotic and abiotic factors are fully described in Table 1.

| **Rank** | **Model components predicting median size of trout** | **df** | **logLik** | **AICc** | **delta AICc** | **weight** |
| --- | --- | --- | --- | --- | --- | --- |
| 1 | YOY + Trout_ab + Hab_size_cascade + T_winter + T_summer | 7 | -149.13 | 314.92 | 0 | 0.14 |
| 2 | YOY + Trout_ab + Salamander_ab + Hab_size_cascade + T_winter + T_summer | 8 | -147.91 | 315.33 | 0.41 | 0.12 |
| 3 | site + YOY + Trout_ab + Hab_side_channel + T_winter + T_summer | 8 | -148.05 | 315.61 | 0.68 | 0.1 |
| 4 | YOY + Trout_ab + Salamander_ab + T_winter + T_summer | 7 | -149.47 | 315.61 | 0.69 | 0.1 |
| 5 | site + YOY + Trout_ab + Salamander_ab + T_winter + T_summer | 8 | -148.26 | 316.04 | 1.12 | 0.08 |
| 6 | YOY + Trout_ab + Salamander_ab + T_winter + T_summer + Q_max | 8 | -148.29 | 316.09 | 1.17 | 0.08 |
| 7 | site + Trout_ab + Hab_side_channel + T_winter + T_summer | 7 | -149.77 | 316.21 | 1.28 | 0.07 |
| 8 | YOY + Trout_ab + Hab_size_cascade + T_winter + T_summer + Q_max | 8 | -148.45 | 316.41 | 1.48 | 0.07 |
| 9 | YOY + Trout_ab + Salamander_ab + Hab_size_cascade + T_winter + T_summer + Q_max | 9 | -147.01 | 316.51 | 1.59 | 0.06 |
| 10 | site + YOY + Trout_ab + Salamander_ab + T_winter + T_summer + Q_max | 9 | -147.04 | 316.58 | 1.65 | 0.06 |
| 11 | YOY + Salamander_ab + T_winter + T_summer + Q_max | 7 | -150.08 | 316.83 | 1.9 | 0.06 |
| 12 | site + Trout_ab + Hab_side_channel + T_winter + T_summer + Q_max | 8 | -148.68 | 316.86 | 1.94 | 0.05 |

**Table S11** Top-supported models (Akaike information criterion corrected for small sample sizes AICc difference among models < 2) and their performance relating body size (median length) of Coastal Giant Salamander and both biotic and abiotic factors (f) in Mack Creek, Oregon Cascades. There were 16,950 candidate models (2^f^) tested after correcting for multicollinearity and missing data. Best-supported models included both density-dependent (abundance of animals) and density-independent (Q = flow; T = temperature; H = habitat size) factors. Biotic and abiotic factors are fully described in Table 1.

| **Rank** | **Model components predicting median size of trout** | **df** | **logLik** | **AICc** | **delta AICc** | **weight** |
| --- | --- | --- | --- | --- | --- | --- |
| 1 | site + Salamander_ab + Hab_size_cascade + T_winter + T_summer + Q_var_low | 8 | -126.95 | 273.4 | 0 | 0.6 |
| 2* | site + Salamander_ab + Hab_size_cascade + T_winter + T_summer + Q_var_low + Q_ratio_max | 9 | -126.54 | 275.57 | 2.17 | 0.2 |
| 3* | site + Salamander_ab + Hab_size_cascade + T_winter + T_summer + Q_var_low + Q_reversal | 9 | -126.56 | 275.62 | 2.22 | 0.2 |

*Models ranked as second and third were also considered and averaged as the best supported as AICc values were very close to the delta AICc threshold.

1. **R scripts used for the trend analysis and model selection procedure**

**6.1 Bootstrapped Mann-Kendall Trend Test with Bias Corrected Prewhitening**

**R code to create Tables S1-9 (data = “trends_covariates.csv”, “trends_length_mm.csv”)**

#Trends in length or covariates

setwd("U:/Your working directory….")

library(modifiedmk)

master=read.csv("trends_covariates.csv or trends_length_mm**.csv** ", header=TRUE)

for (i in 2:ncol(master)) {

trend<-pbmk(master[,i], nsim=2000, pw="Hamed")

trend_output<-print(paste(trend))

write.table(trend_output, file = "results_trends_covariates.csv",

sep = ",", append = TRUE,

col.names = TRUE, row.names = TRUE)

}

#The output vector contains the value of ten metrics in the following order 'Z Value'

#'Sen's Slope', 'S', 'p-value'', Kendall's Tau',

#'BCP Z Value, 'BCP Sen's Slope, 'BCP S', 'BCP Kendall's Tau', and

#'Bootstrapped p-value'

#Note that Sen’s slopes as magnitude of trends are estimated as ‘unit per year’. In tables S1-S9, #these slopes were transformed to ‘mm per decade’

**6.2 Model selection Procedure to predict animal size based on covariates**

**R code to create Tables 2-3, Figure 6 and S10-11 (data = “model_selection.csv”)**

library(rJava)

library(MuMIn)

library(car) # extracts model results

library(carData)

library(sjPlot) # visualizes model results

library(effects)

library(gridExtra)

library(glmulti) # finds the BEST model

library(leaps)

library(flextable) # beautifies tables

library(tidyverse) # provides a lot of useful stuff !!!

library(performance)# checks and compares quality of models

# install.packages("devtools")

#devtools::install_github("r-lib/conflicted")

setwd("U:/Your working directory….")

master <-read.csv("model_selection.csv", header=TRUE)

#z-score covariates to compare model coefficients

master$YOY <- scale(master$YOY)

master$Trout_ab <- scale(master$Trout_ab)

master$Salamander_ab <- scale(master$Salamander_ab)

master$Hab_size_cascade <- scale(master$Hab_size_cascade)

master$Hab_size_pool <- scale(master$Hab_size_pool)

master$Hab_side_channel <- scale(master$Hab_side_channel)

master$T_winter <- scale(master$T_winter)

master$T_summer <- scale(master$T_summer)

master$Q_var_low <- scale(master$Q_var_low)

master$Q_reversal <- scale(master$Q_reversal)

master$Q_max <- scale(master$Q_max)

master$Q_ratio_min <- scale(master$Q_ratio_min)

master$Q_ratio_max <- scale(master$Q_ratio_max)

###### trout model that predicts median size (excluding covariates that showed collinearity – see Table 1#####

global.model.CCT <- glm(trout_50th ~ site

+ YOY

+ Trout_ab

+ Salamander_ab

+ Hab_size_cascade

+ Hab_size_pool

+ Hab_side_channel

+ T_winter

+ T_summer

+ Q_var_low

+ Q_reversal

+ Q_max

+ Q_ratio_min

+ Q_ratio_max

, data=master)

CCT.model <- glmulti(global.model.CCT, level = 1, crit="aicc")

weightable(CCT.model)

#Inspect output and keep top-supported models based on weightable (use AICc difference of 2).

#for trout we average top-12 models accordingly

m_1 <- glm(trout_50th ~ 1 + YOY + Trout_ab + Hab_size_cascade + T_winter + T_summer, data=master)

m_2 <- glm(trout_50th ~ 1 + YOY + Trout_ab + Salamander_ab + Hab_size_cascade + T_winter + T_summer, data=master)

m_3 <- glm(trout_50th ~ 1 + site + YOY + Trout_ab + Hab_side_channel + T_winter + T_summer, data=master)

m_4 <- glm(trout_50th ~ 1 + YOY + Trout_ab + Salamander_ab + T_winter + T_summer, data=master)

m_5 <- glm(trout_50th ~ 1 + site + YOY + Trout_ab + Salamander_ab + T_winter + T_summer, data=master)

m_6 <- glm(trout_50th ~ 1 + YOY + Trout_ab + Salamander_ab + T_winter + T_summer + Q_max, data=master)

m_7 <- glm(trout_50th ~ 1 + site + Trout_ab + Hab_side_channel + T_winter + T_summer, data=master)

m_8 <- glm(trout_50th ~ 1 + YOY + Trout_ab + Hab_size_cascade + T_winter + T_summer + Q_max, data=master)

m_9 <- glm(trout_50th ~ 1 + YOY + Trout_ab + Salamander_ab + Hab_size_cascade + T_winter + T_summer + Q_max, data=master)

m_10 <- glm(trout_50th ~ 1 + site + YOY + Trout_ab + Salamander_ab + T_winter + T_summer + Q_max, data=master)

m_11 <- glm(trout_50th ~ 1 + YOY + Salamander_ab + T_winter + T_summer + Q_max, data=master)

m_12 <- glm(trout_50th ~ 1 + site + Trout_ab + Hab_side_channel + T_winter + T_summer + Q_max, data=master)

#Model average of top supported models (difference in AICc <2)

f.aveCCT <- model.avg(m_1, m_2, m_3, m_4, m_5, m_6, m_7, m_8, m_9, m_10, m_11, m_12)

summary(f.aveCCT)

#Details of top supported models

weightable(CCT.model)[1:12,] %>%

regulartable() %>% # beautifying tables

autofit()

#Effect size of average model

plot(f.aveCCT, type = "s")

Confint(f.aveCCT, level = 0.95)

###### salamander model that predicts median size #####

global.model.CGS <- glm(salamander_50th ~ site

+ YOY

+ Trout_ab

+ Salamander_ab

+ Hab_size_cascade

+ Hab_size_pool

+ Hab_side_channel

+ T_winter

+ T_summer

+ Q_var_low

+ Q_reversal

+ Q_max

+ Q_ratio_min

+ Q_ratio_max

, data=master)

CGS.model <- glmulti(global.model.CGS, level = 1, crit="aicc")

weightable(CGS.model)

#Inspect output and keep top-supported models based on weightable (use AICc difference of 2)

m_s1 <- glm(salamander_50th ~ 1 + site + Salamander_ab + Hab_size_cascade + T_winter + T_summer + Q_var_low, data=master)

m_s2 <- glm(salamander_50th ~ 1 + site + Salamander_ab + Hab_size_cascade + T_winter + T_summer + Q_var_low + Q_ratio_max, data=master)

m_s3 <- glm(salamander_50th ~ 1 + site + Salamander_ab + Hab_size_cascade + T_winter + T_summer + Q_var_low + Q_reversal, data=master)

#Model average of top supported models (difference in AICc <2); however models 2 and 3 seems very close to model 1 based on the IC profile plot

#therefore, we averaged models 1, 2 and 3

f.aveCGS <- model.avg(m_s1, m_s2, m_s3)

summary(f.aveCGS)

#Details of top supported models

weightable(CGS.model)[1:3,] %>%

regulartable() %>% # beautifying tables

autofit()

#Effect size of average model

plot(f.aveCGS, type = "s")

Confint(f.aveCCT, level = 0.95)
